# Dps-dependent *in vivo* mutation enhances long-term host adaptation in *Vibrio cholerae*

**DOI:** 10.1371/journal.ppat.1011250

**Published:** 2023-03-16

**Authors:** Mei Luo, Guozhong Chen, Chunrong Yi, Baoshuai Xue, Xiaoman Yang, Yao Ma, Zixin Qin, Jin Yan, Xiaoyun Liu, Zhi Liu

**Affiliations:** 1 Key Laboratory of Molecular Biophysics of the Ministry of Education, Department of Biotechnology, College of Life Science and Technology, Huazhong University of Science and Technology, Wuhan, Hubei, China; 2 Department of Microbiology and Infectious Disease Center, School of Basic Medical Sciences, Peking University Health Science Center, Beijing, China; 3 NHC Key Laboratory of Medical Immunology, Peking University, Beijing, China; 4 Key Laboratory of Zoonosis, Ministry of Education, College of Veterinary Medicine, Jilin University, Changchun, China; University of North Carolina at Chapel Hil, UNITED STATES

## Abstract

As one of the most successful pathogenic organisms, *Vibrio cholerae* (*V*. *cholerae*) has evolved sophisticated regulatory mechanisms to overcome host stress. During long-term colonization by *V*. *cholerae* in adult mice, many spontaneous nonmotile mutants (approximately 10% at the fifth day post-infection) were identified. These mutations occurred primarily in conserved regions of the flagellar regulator genes *flrA*, *flrC*, and *rpoN*, as shown by Sanger and next-generation sequencing, and significantly increased fitness during colonization in adult mice. Intriguingly, instead of key genes in DNA repair systems (*mutS*, *nfo*, *xthA*, *uvrA*) or ROS and RNS scavenging systems (*katG*, *prxA*, *hmpA*), which were generally thought to be associated with bacterial mutagenesis, we found that deletion of the cyclin gene *dps* significantly increased the mutation rate (up to 53% at the fifth day post-infection) in *V*. *cholerae*. We further determined that the *dps*^D65A^ and *dps*^F46E^ point mutants showed a similar mutagenesis profile as the Δ*dps* mutant during long-term colonization in mice, which strongly indicated that the antioxidative function of Dps directly contributes to the development of *V*. *cholerae* nonmotile mutants. Methionine metabolism pathway may be one of the mechanism for Δ*flrA*, Δ*flrC* and Δ*rpoN* mutant increased colonization in adult mice. Our results revealed a new phenotype in which increased fitness of *V*. *cholerae* in the host gut via spontaneous production nonmotile mutants regulated by cyclin Dps, which may represent a novel adaptation strategy for directed evolution of pathogens in the host.

## Author summary

Pathogen–host coevolution during long-term infection is the main strategy by which pathogens adapt to the host environment and coexist with humans. The host usually initiates a series of immune responses against the pathogen by sensing pathogen-associated molecular patterns, including flagella and lipopolysaccharide. Among them, the reactive oxygen/nitrogen species (ROS/RNS) produced by the host can quickly lead to loss of the cytoplasmic membrane and DNA damage in pathogens, and directly kill the pathogens or inhibit their growth. *V*. *cholerae*, one of the oldest pathogenic enteric bacteria, has evolved elaborate regulatory mechanisms in response to ROS, including inhibition of ROS production by Dps and scavenging of ROS by PrxA, KatG and KatB. Here, we report a novel adaptation strategy in which *V*. *cholerae* produces high-frequency spontaneous nonmotile mutants during long-term colonization in the adult mouse gastrointestinal tract. In the nonmotile mutants, we identified that the hotspots for the spontaneous mutations are the flagellar regulatory genes *flrA*, *flrC* and *rpoN*. These nonmotile mutants exhibited enhanced host adaptation in adult mice, possibly through distinct mechanisms. Further studies revealed that only cyclin Dps, not the classic DNA repair systems or ROS and RNS scavenging systems, were closely involved in mutagenesis in the nonmotile mutants. These results may represent a novel adaptation strategy for the directed evolution of pathogens in the host. Our results might explain why many clinical isolates are nonmotile in cholera outbreak areas such as Haiti and Nepal. The spontaneously generated nonmotile mutants of *V*. *cholerae* during long-term colonization exhibit increased adaptation in the host and the potential to become epidemic strains, which warrants the attention of epidemiologists and bacteriologists.

## Introduction

In recent regional cholera outbreaks in Haiti and Africa, the severe watery diarrheal disease caused by *Vibrio cholerae* (*V*. *cholerae*) still exhibited high morbidity and mortality [1–3]. *V*. *cholerae* is a noninvasive pathogen that colonizes epithelial surface of the small intestine (SI) after oral ingestion of contaminated food or water [4]. During *V*. *cholerae* epithelium colonization, the bacteria must penetrate the mucosal barrier on the surface of intestinal villi mediated by the single polar flagellum [5,6]. Karl E. Klose et al. performed extensive research on flagellar regulation in *V*. *cholerae* and found that flagellar biogenesis involves a four-tiered transcriptional hierarchy [7]. The key flagellar regulatory genes include *flrA* (class I) and *flrC* (class II), and the functions of FlrA and FlrC require the assistance of another nonadjacent regulatory protein, RpoN [8]. The class III gene, *flaA*, encodes an essential “core” flagellin [9], and class IV genes encode other flagellin proteins and motor components [10]. We have reported that *V*. *cholerae* breaks the flagellum during mucus penetration and enhances the expression of virulence genes by suppressing the quorum sensing gene *hapR* [11]. The lack of flagellum can also facilitate infection by preventing the adhesion of host innate immunity proteins [12], and flagellar gene mutations were found in clinical isolates from cholera outbreak areas in Haiti and Nepal [13]. However, the literature overwhelmingly demonstrates a lack of flagellum-related genes attenuation of *V*. *cholerae* colonization in infant mice [8,11,14]. This seems to be a paradoxical phenomenon.

Bacterial flagella are potent antigens that are recognized by host TLR5 receptors to rapidly activate innate immunity and produce proinflammatory cytokines, reactive oxygen species (ROS) and reactive nitrogen species (RNS), which cause bacterial DNA damage and cell death [15–18]. *V*. *cholerae* has the ability to cope with host-produced ROS and RNS by upregulating OxyR-activated expression of *dps*, *katG*, *prxA* or AphB-mediated expression of *ohrA* in response to ROS [19–21] and NorR-mediated expression of *nnrS* and *hmpA* in response to RNS [22] or by promoting bacterial mutation frequency to improve environmental adaptability. Among them, Dps is a conserved multifunctional cyclin protein, in addition to its role in ROS resistance, protecting bacterial cells from various stresses, such as oxidative stress, UV, iron and copper toxicity, and acid and base shock [23,24], and is prevalent in prokaryotic cells with homologs identified in over 300 species of bacteria [25].

Bacteria have also developed several sophisticated defense mechanisms against DNA damage, which have been broadly classified into several pathways: the mismatch DNA repair pathway (MMR), base excision repair pathway (BER), nucleotide excision repair pathway (NER) and SOS system. Intriguingly, the seventh pandemic of cholera has spread from the Bay of Bengal in at least three independent but overlapping waves, and the El Tor isolates of the 7th pandemic have a very consistent rate of SNPs (single nucleotide polymorphisms, SNPs) accumulation (3.3 SNPs per year) in the *V*. *cholerae* core genome [1]. Limited but detectable diversity at the level of zero to three single nucleotide variants was observed in patients from Bangladesh and Haiti [26]. Forty-five high-quality SNPs (hqSNPs) from 108 genomes have been uncovered in isolates from Haiti and Nepal, two of which were the flagellar structural protein genes *flaE* and *flgK* (two isolate genomes have hqSNPs in *flaE*, seven in *flgK*) [13].

In this study, we reported that *V*. *cholerae* produced high-frequency spontaneous nonmotility-related mutants during passage through the adult mouse gastrointestinal tract. We discovered an unexpected and previously unrecognized feature of *V*. *cholerae*, that is, the spontaneously generated nonmotile mutants exhibited increased ability of adult mouse intestinal colonization. Furthermore, we showed that the defective ROS resistance function of Dps was responsible for the spontaneous generation of nonmotile mutants.

## Results

### *V*. *cholerae* produces spontaneous nonmotility-related mutations during long-term colonization in adult mice

**Fig 1:**
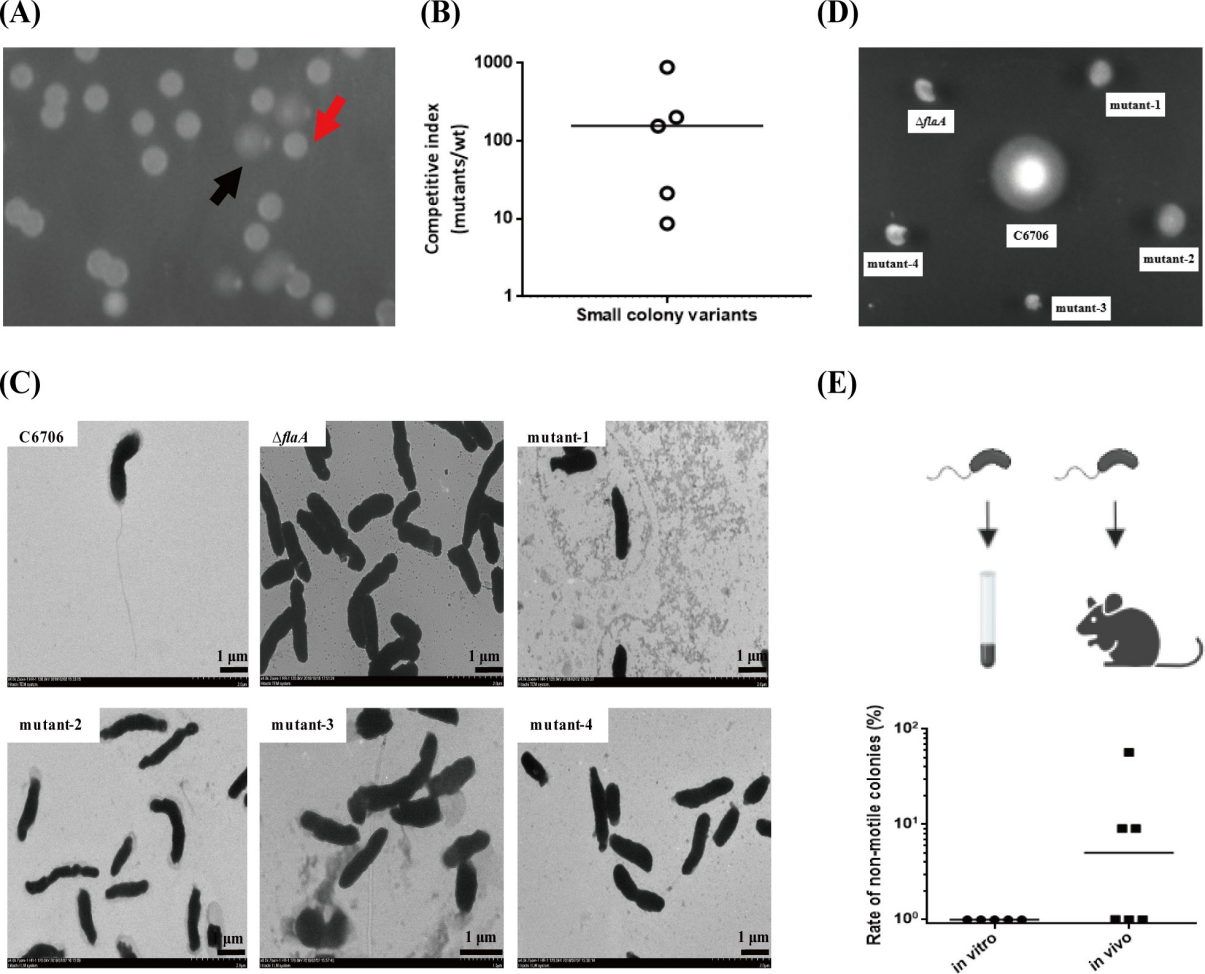
*V*. *cholerae* produces spontaneous nonmotility-related mutations during long-term colonization in adult mice. **(A) Different morphological *V*. *cholerae* were found in the intestine of adult mice.** We collected the fecal pellets of adult mice at the fifth day post-infection and plated on plates. Unexpectedly found the small, rugose, opacity and dense colony (red arrow), which was different morphology from wild-type *V*. *cholerae* large, smooth and transparent colony (black arrow). **(B) Adult mice competition assay of small colony variants.** We pick several small colony variants from different individual mouse and mixed as a sample, 10^8^ cells of wild-type and small colony variants were mixed in a 1:1 ratio and intragastrically administered to CD-1 adult mice. Fecal pellets were collected from each mouse at the fifth day after infection and plated on selective plates. The competitive index (CI) was calculated as the ratio of small colony variants to wild-type colonies normalized to the input ratio. Horizontal line: median CI. **(C) Electron micrographs of small colony variants.** Bacteria were harvested in mid-logarithmic growth and prepared for electron microscopy. Bars represent 1 μm, C6706, flagellum positive control. Δ*flaA*, flagellum negative control. Mutant-1-4, small colony variants. **(D) Motility phenotype of small colony variants.** Bacteria were inoculated into 0.3% agar LB plates and incubated at 37°C for 8 h. C6706, motility positive control. Δ*flaA*, motility negative control. Mutant-1-4, small colony variants. **(E) Rate of nonmotile *V*. *cholerae in vitro* and *in vivo* culture.** Wild-type C6706 were inoculated in 5 mL LB anaerobic test tubes and incubated anaerobically at 37°C for 5 days, and plated onto selective plates (*in vitro* culture). Bacteria were intragastrically administered to CD-1 adult mice, fecal pellets were collected from each mouse at the fifth day after infection and plated onto selective plates (*in vivo* culture). At the indicated time points, one hundred *V*. *cholerae* colonies per sample were picked and the motility was detected in 0.3% agar LB plates. Rate of nonmotile colonies were calculated as the ratio of nonmotile mutant colonies to all colonies per sample. Horizontal line: median. The illustration was created with BioRender.com.

To mimic the actual *V*. *cholerae* infection cycle, a sustained adult mouse colonization model was constructed, in which the time periods of *V*. *cholerae* colonization were similar to those of actual infection in the human intestine [22]. Surprisingly, we often found two morphologies of *V*. *cholerae* colonies in plates coated with late colonized (5–7 days post-infection) mouse feces. One was the classic large, smooth and transparent *V*. *cholerae* colony morphology (as shown by the black arrow in Fig 1A), and the other was the small, rugose, opaque and dense colony morphology (as shown by the red arrow in Fig 1A), which was confirmed as *V*. *cholerae* by 16S sequencing. We speculated that small colony variants may have a colonization advantage in adult mice. To test this hypothesis, several small colony variants from different individual mouse experiments were picked and mixed for the mouse competition assay. The results showed that small colony variants enhanced colonization ability in adult mice, with an approximately 100-fold advantage over the wild-type at 5 days post inoculation (Fig 1B). Electron microscopy verified that those strains lacked a flagellum (Fig 1C), which was further validated by a motility assay on 0.3% agar LB plate (Fig 1D). As expected, no nonmotile mutants were found in the *in vitro* culture experiment, whereas approximately 10% of the recovered bacteria exhibited nonmotility after 5 days of intestinal colonization in adult mice (Fig 1E). Taken together, these results suggested that *V*. *cholerae* may enhance adult mouse adaptation by spontaneously producing nonmotile mutants.

### High impact variants in nonmotile mutants are located on the flagellum regulator genes *flrA*, *flrC* and *rpoN*

**Fig 2:**
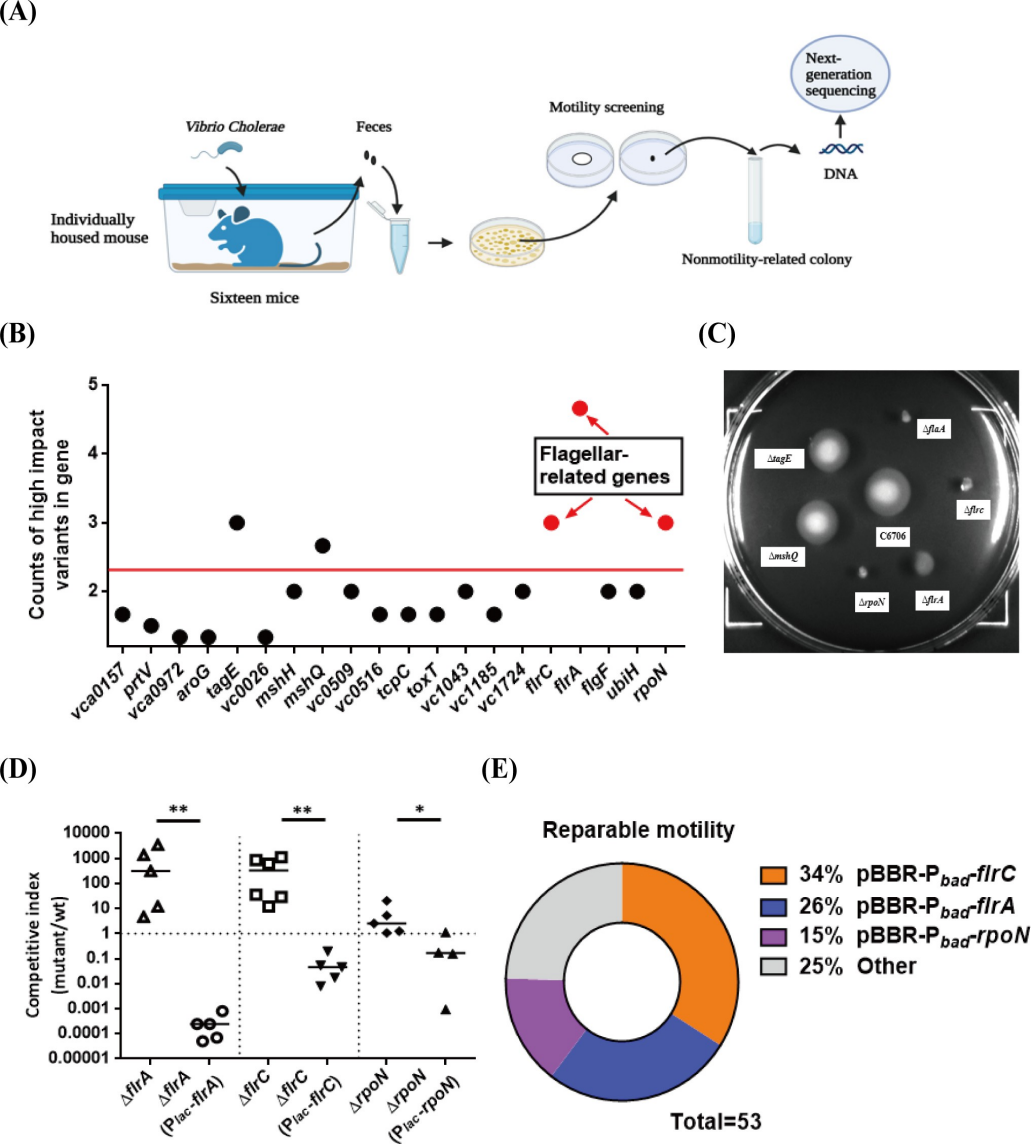
High impact variants in nonmotile mutants are located on the flagellum regulator genes *flrA*, *flrC* and *rpoN*. **(A) Schematic experimental overview for next-generation sequencing.** Wild-type C6706 were intragastrically administered to 16 CD-1 adult mice. A single-cage single-mouse infection experiment were performed to avoid a cage effect of mutants’ collection. Fecal pellets were collected from each mouse at the fifth day post-infection, one hundred *V*. *cholerae* colonies from one mouse were randomly selected for motility screening in 0.3% agar LB plates. To avoid picking siblings of the same bacterium, we picked only 3–4 nonmotile colonies per mouse for next-generation sequencing. The illustration was created with BioRender.com. **(B) High impact variants of *V*. *cholerae* from intestine of adult mice.** Fifty-three nonmotile mutants from 16 mice were chosen for DNA purification and next-generation sequencing, the genes of 20 High impact variants were showed. **(C) Motility phenotype of five High impact knockout mutants.** Bacteria were inoculated into 0.3% agar LB plates and incubated at 37°C for 8 h. C6706, motility positive control. Δ*flaA*, motility negative control. **(D) Adult mice competition assay of Δ*flrA*, Δ*flrC*, Δ*rpoN*.** 10^8^ cells of wild-type and knockout or complemented strain were mixed in a 1:1 ratio and intragastrically administered to CD-1 adult mice, the competitive index (CI) of the fifth day after infection was calculated as the ratio of mutant to wild-type colonies normalized to the input ratio. Horizontal line: median CI. Significance was determined by Mann Whitney test, *p*-value: *, < 0.05, **, < 0.01. **(E) Reparable motility phenotype of nonmotile mutants derived from C6706 by *flrA*, *flrC*, *rpoN* genes.** The pBBR-P_*bad*_-*flrA*, pBBR-P_*bad*_-*flrC* and pBBR-P_*bad*_-*rpoN* plasmids were constructed to complement the motility phenotype of fifty-three nonmotile mutants derived from C6706. Detection of reparable motility by 0.3% agar LB plates.

To identify the gene(s) contributing to the deficiency of *V*. *cholerae* motility, we detected the location of the mutations using whole genome next-generation sequencing. Because mice are coprophages, we used a single-cage single-mouse infection experiment to ensure that the *V*. *cholerae* isolates recovered from each mouse were derived from a single mutation event. One hundred *V*. *cholerae* colonies per mouse were picked, and the motility was checked in 0.3% agar LB plates. To avoid picking siblings of the same bacterium, only 3–4 nonmotile colonies per mouse were collected for subsequent tests. In total, 53 nonmotile mutants from 16 mice were chosen for sequencing following the experimental scheme shown in Fig 2A. The sequencing data obtained were analyzed by SnpEff [27] and revealed that High impact variants were mainly located in *flrA*, *flrC*, *rpoN*, *tagE*, and *mshQ* (Fig 2B and S2 Table).

As previously reported, *flrA*, *flrC* and *rpoN* are the classic flagellar regulatory genes of *V*. *cholerae* [7,8]. Clean knockout of the *flrA*, *flrC* and *rpoN* genes resulted in a nonmotile phenotype in 0.3% agar LB plates (Fig 2C). We confirmed that these three mutants showed a colonization advantage in an adult mouse long-term colonization model, and chromosomal complementation of these genes at the *lacZ* locus decreased the colonization ability of the mutants (Fig 2D). The *in vitro* growth competition assays results showed that Δ*flrA*, Δ*flrC* and Δ*rpoN* mutants exhibited a growth disadvantage compared to the wild-type, suggesting that the *in vivo* colonization advantage may be unrelated to growth (S1 Fig). Meanwhile, we found that deletion of the essential “core” flagellin gene *flaA* (not flagellated, nonmotile) [9] attenuated colonization in adult mice and implied that the colonization advantage of Δ*flrA*, Δ*flrC* and Δ*rpoN* was not associated with *V*. *cholerae* nonmotility (S2A Fig). However, we did not observe a nonmotile phenotype or colonization advantage for the other two candidate genes, namely, *tagE* and *mshQ* (Figs 2C and S2B). It is possible that the other mutations that occurred in the *tagE* and *mshQ* mutant isolates led to nonmotile phenotype and enhanced colonization.

To confirm that mutations in the *flrA*, *flrC* and *rpoN* genes were the major mutated loci in the above nonmotile mutants, we performed complementation experiments. The pBBR-P_*bad*_-*flrA*, pBBR-P_*bad*_-*flrC*, and pBBR-P_*bad*_-*rpoN* plasmids were introduced into all 53 nonmotile mutants to test if they could complement the motility phenotype. The results showed that the motility of 14 mutants was restored by the pBBR-P_*bad*_-*flrA* plasmid (26%), and that of 18 and 8 mutants was restored by pBBR-P_*bad*_-*flrC* plasmid (34%) and pBBR-P_*bad*_-*rpoN* plasmid (15%), respectively (Fig 2E). Our data suggested that *V*. *cholerae* may enhance colonization in adult mice by generating spontaneous mutations mainly in the flagellar regulator genes *flrA*, *flrC* and *rpoN*.

### Nonmotility-related mutation frequency is *dps-*dependent in *V*. *cholerae*

**Fig 3:**
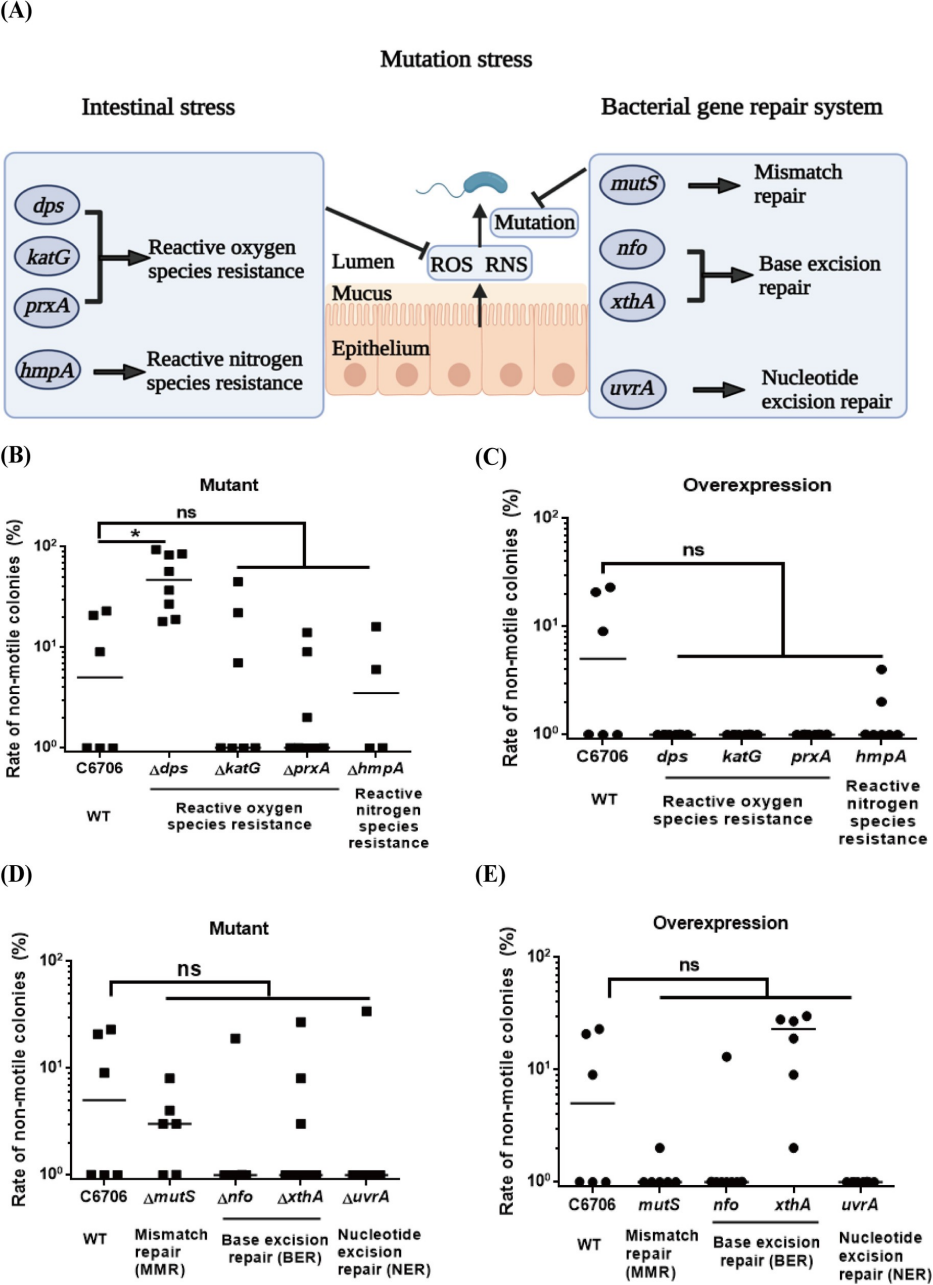
Nonmotility-related mutation frequency is *dps-*dependent in *V*. *cholerae*. **(A) Schematic experimental overview.** The illustration was created with BioRender.com. **(B) Effect of *V*. *cholerae* defective in ROS or RNS detoxification genes on the rate of nonmotile colonies *in vivo*.** *V*. *cholerae* defective in ROS (Δ*dps*, Δ*katG*, Δ*prxA*) or RNS (Δ*hmpA*) detoxification genes were individually intragastrically inoculated into CD-1 mice treated with antibiotics cocktail. Fecal pellets were collected at the fifth day post-infection, and plated on selective plates. One hundred *V*. *cholerae* colonies from one mouse were randomly selected for motility screening in 0.3% agar LB plates. Rate of nonmotile colonies were calculated as the ratio of nonmotile mutant colonies to all colonies per sample. Horizontal line: median. Significance was determined by Kruskal-Wallis test, *p*-value: ns, not significant, *, < 0.05. **(C) Effect of *V*. *cholerae* overexpression in ROS or RNS detoxification genes on the rate of nonmotile colonies *in vivo*.** Wild-type *V*. *cholerae* containing pACYC-P_*bad*_-*dps*, pACYC-P_*bad*_-*katG* or pACYC-P_*bad*_*-prxA* plasmid for overexpression in ROS detoxification, and pACYC-P_*bad*_-*hmpA* plasmid for overexpression in RNS detoxification were individually intragastrically inoculated into CD-1 mice. Fecal pellets were collected at the fifth day post-infection, and plated on selective plates. One hundred *V*. *cholerae* colonies from one mouse were randomly selected for motility screening in 0.3% agar LB plates. Rate of nonmotile colonies were calculated as the ratio of nonmotile mutant colonies to all colonies per sample. Horizontal line: median. Significance was determined by Kruskal-Wallis test, *p*-value: ns, not significant. **Effect of *V*. *cholerae* defective (D) and overexpression (E) in bacterial gene repair system on the rate of nonmotile colonies *in vivo*.** Horizontal line: median. Significance was determined by Kruskal-Wallis test, *p*-value: ns, not significant.

Reactive oxygen species (ROS) and reactive nitrogen species (RNS) can effectively induce gut bacterial mutation, which is normally repaired by the DNA repair system to maintain inheritance stability in bacteria (Fig 3A). As previously reported, Dps, KatG, and PrxA are the major effector proteins for ROS inhibition and scavenging, HmpA is a key protein for RNS scavenging in *V*. *cholerae*, and its transcriptional expression can be effectively stimulated by host environmental signals [21,28]. We independently knocked out *dps*, *katG*, *prxA* and *hmpA* and checked their effect on nonmotile mutants produced in adult mice. Intriguingly, deletion of only *dps* but not *katG*, *prxA* and *hmpA* significantly increased abundance of nonmotile mutants in the recovered *V*. *cholerae* population from mouse feces (Fig 3B). We complemented Δ*dps* by introducing a knock-in sequence containing the *dps* gene (*dps*^C^) into the Δ*dps* knockout mutant. The rate of nonmotile mutants of *dps*^C^ in adult mouse fecal pellets was significantly lower than that of the Δ*dps* mutant (S3A Fig). Interestingly, our previous research showed that the expression of the *dps* gene in *V*. *cholerae* was the most significant in response to H_2_O_2_ exposure [21]. Together with our results, these findings suggest that more studies are needed to explore the function of Dps in *V*. *cholera* pathogenesis. However, overexpression of all the ROS and RNS resistance genes above almost completely eliminated the generation of nonmotile mutants of wild-type *V*. *cholerae* (Fig 3C), which suggested that scavenging ROS or RNS stress in *V*. *cholerae* can reduce DNA damage and mutagenesis.

To determine whether DNA repair systems are required for flagellum-regulatory adaptation in adult mice, we use *mutS*, which encodes a gene product that initially binds to a mismatch, as a representative gene required for MMR [29]; *nfo* and *xthA* encode apurinic/apyrimidininc (AP) endonucleases, as representative genes for BER [28]; *uvrA* encodes the protein that recognizes and binds to the damaged DNA site, as a representative gene for NER [30]; and *lexA* encodes a regulator protein for the SOS system [31]. Δ*lexA* exhibited a growth defect in M9 minimal medium (M9 salts plus 2 mM MgSO_4_, 0.1 mM CaCl_2_, and 0.2% glucose as the sole carbon source, S3B Fig), and no further research could be performed.

Neither deletion nor overexpression of a functional gene of the MMR, BER or NER system was associated with nonmotile mutant production *in vivo* (Fig 3D and 3E); however, the lack of a functional gene of the MMR system but not the BER and NER systems enhanced the mutation rate for *V*. *cholerae in vivo*, as shown by a rifampicin resistance assay (S3C Fig). Our data suggested that the lack of a functional Dps increases the frequency of nonmotile mutants in *V. cholerae*.

### ROS detoxification function of Dps contributes to the nonmotility-related mutation frequency in *V*. *cholerae*

Dps, initially named DNA-binding protein from starved cells (Dps), is a conserved multifunctional protein protecting bacterial cells from various stresses, such as oxidative stress, UV, iron and copper toxicity, and acid and base shock [32,33]. We found that a lack of functional Dps increased the frequency of nonmotile mutant production; however, Δ*dps* itself did not affect *V*. *cholerae* motility on 0.3% agar LB plates (S3D Fig). Upon exposure to higher levels of H_2_O_2_, an elevated mutation rate was detected in both wild-type C6706 and Δ*dps* (S3E Fig). However, compared to the wild-type, the change in mutation rate in Δ*dps* mutants had no statistical significance either *in vitro* or *in vivo* (S3E and S3F Fig). These results showed that Δ*dps* had no positive effect on the frequency of gene mutation. The multifaceted protective function of Dps is mostly conferred by DNA binding, iron sequestration, and its ferroxidase activity [24]. We speculated that the Dps-related protection of DNA from mutation may be related to the function of detoxification of oxidative stress and DNA binding.

**Fig 4:**
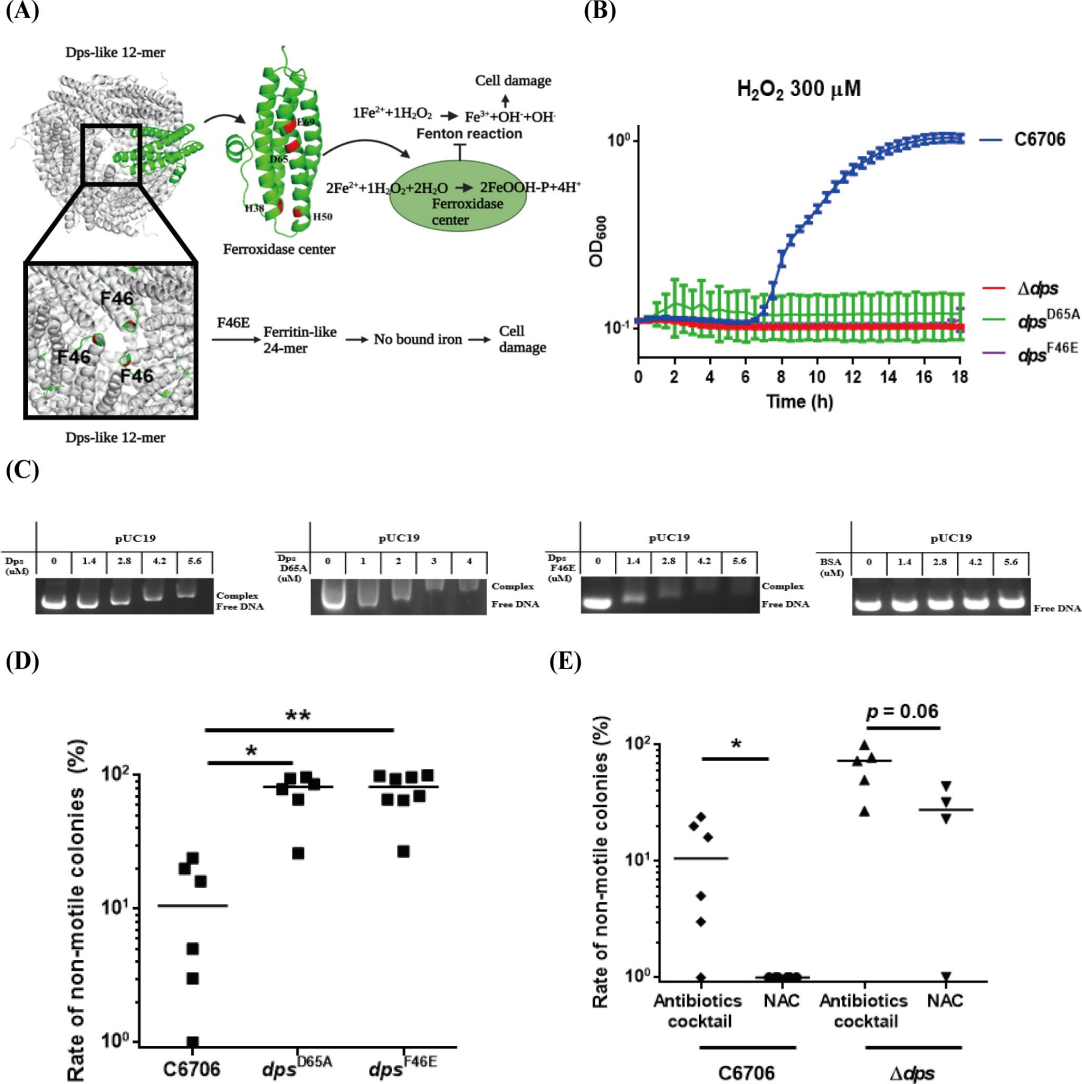
ROS detoxification function of Dps contributes to the nonmotility-related mutation frequency in *V*. *cholerae*. **(A) Dps protein structure of ROS detoxification.** Structural model of Dps generated from the crystal structure of the *V*. *cholerae* N16961 (PDB entry 3IQ1) was showed. The monomer of the Dps-like 12-mer assemblies was showed by green (top left), which the ferroxidase center amino acid residues (H38, H50, D65, E69) were highlighted in red. Dps protects DNA with ferroxidase center by greatly ameliorating the potentially lethal combination of Fe^2+^ and H_2_O_2_. F46 was colored red, residues of AB loop were colored green (bottom left). A mutation at F46 might generate a 24-mer from Dps, the conformations of ferroxidation site residues were altered and no iron was bound due to disruption of stacking interactions with F46, which may result in Dps ROS detoxification function deficiency and consequent cell damage. **(B) Growth of wild-type and *dps* point mutations under ROS stress.** Exponentially growing cultures of wild-type C6706 (blue) and Δ*dps* (red), *dps*^D65A^ (green) and *dps*^F46E^ (purple) mutants were grown in LB with 300 μM H_2_O_2_. The recovery and growth of each strains were monitored over time. The averages of 4 experiments were showed for each strains. **(C) Binding of Dps to supercoiled plasmid pUC19.** Different concentration wild-type Dps or D65A, F46E Dps mutants protein were individually incubated with 0.6 pM of supercoiled plasmid pUC19 (in 50 mM MOPS buffer pH 7.0, containing 50 mM NaCl). BSA, negative control. **(D) Rate of nonmotile mutants in *dps***^**D65A**^ **and *dps***^**F46E**^ **mutations in adult mice intestine.** Δ*dps*, *dps*^D65A^ and *dps*^F46E^ mutants were individually intragastrically inoculated into CD-1 mice treated with antibiotics cocktail. Fecal pellets were collected at the fifth day post-infection, and plated on selective plates. One hundred *V*. *cholerae* colonies from one mouse were randomly selected for motility screening in 0.3% agar LB plates. Rate of nonmotile colonies were calculated as the ratio of nonmotile mutant colonies to all colonies per sample. Horizontal line: median. Significance was determined by Kruskal-Wallis test, *p*-value: *, < 0.05, **, < 0.01. **(E) Rate of nonmotile mutants in wild-type C6706 and Δ*dps* in adult mice intestine with or without ROS.** Bacteria were intragastrically administered to CD-1 adult mice treated with antibiotics cocktail (ROS+) or NAC (ROS-), fecal pellets were collected from each mouse at the fifth day post-infection, and plated onto selective plates. One hundred *V*. *cholerae* colonies per mouse were randomly selected for motility screen in 0.3% agar LB plates. Rate of nonmotile colonies were calculated as the ratio of nonmotile mutant colonies to all colonies per sample. Horizontal line: median. Significance was determined by Mann Whitney test, *p*-value: *, < 0.05.

The ferroxidase function of Dps has been most well studied in bacteria, and the ferroxidase center D78 has been found to be an important amino acid residue for DNA protection in *Escherichia coli* (*E*. *coli*) [32]. Mutating a residue F47 to E47 of Dps altered its structure from a canonical 12-mer to a ferritin-like 24-mer in *Mycobacterium smegmatis* (*M*. *smegmatis*) [34]. According to amino acid sequence alignments of representative Dps proteins, *V*. *cholerae* carries close the homologous residues D65 and F46 (S4 Fig). An illustration of the *V*. *cholerae* Dps protein amino acid residues important for ROS detoxification function was showed in Fig 4A. We created the *dps*^D65A^ and *dps*^F46E^ point mutants, which displayed decreased resistance to H_2_O_2_ (Fig 4B) but retained the DNA binding function (Fig 4C) and exhibited an increased rate of nonmotile mutants in adult mouse intestine compared to the wild-type (Fig 4D). Then, we used N-acetyl-L-cysteine (NAC), a scavenger of reactive oxygen species [35], to remove intestinal ROS in mice. In the NAC-treated mouse model, wild-type C6706 and Δ*dps* (*p* = 0.06) both had a decreased rate of nonmotile mutants compared to that in the antibiotic cocktail-treated mouse model (Fig 4E). As expected, markedly decreased ROS levels were found in the NAC-treated adult mouse small intestine (S5A and S5B Fig). Collectively, these data showed that the ROS detoxification function of Dps is associated with the production of nonmotile mutants *in vivo*.

### Similar mutation characteristics between nonmotile mutants derived from the Δ*dps* mutant or wild-type *V*. *cholerae*

We asked if the nonmotile mutants derived from the Δ*dps* mutant were also mutated in the flagellar regulatory genes. Similarly, the 3 plasmids pBBR-P_*bad*_-*flrA*, pBBR-P_*bad*_-*flrC*, pBBR-P_*bad*_-*rpoN* were used to test whether they could complement the motility phenotype of 51 nonmotile mutants from thirteen mice infected with the Δ*dps* mutant followed by a single-cage single-mouse strategy. The results showed that the 3 plasmids above can restore the motility of a majority of the nonmotile mutants. The motility of 5 mutants was restored by the pBBR-P_*bad*_-*flrA* plasmid (10%), and the motility of 1 mutant and 32 mutants was restored by the pBBR-P_*bad*_-*flrC* plasmid (2%) and pBBR-P_*bad*_-*rpoN* plasmid (63%), respectively (S6 Fig), which suggested that they have similar mutation characteristics in nonmotile mutants derived from the Δ*dps* mutant or wild-type *V*. *cholerae in vivo*.

**Fig 5:**
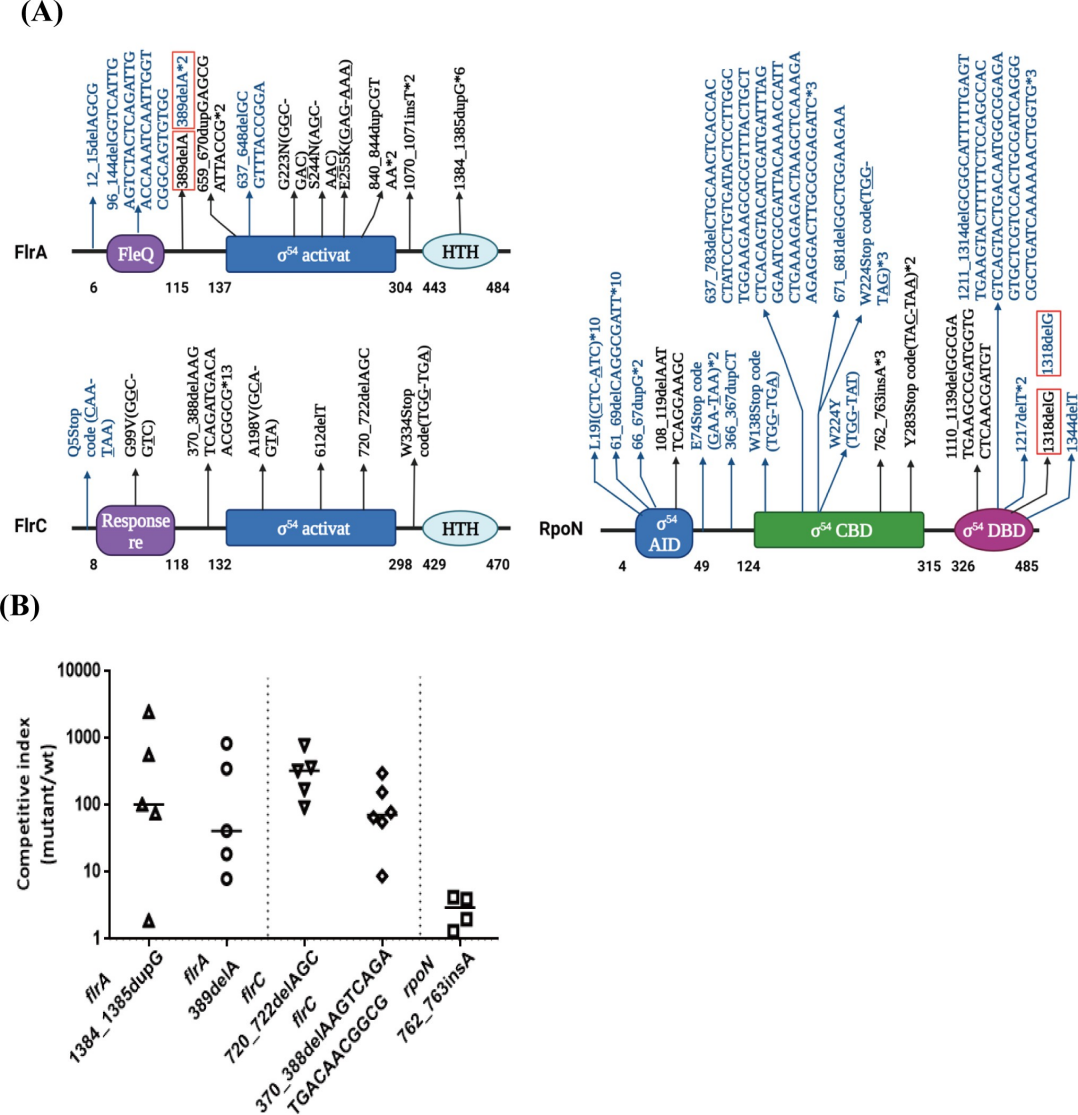
Similar mutation characteristics between nonmotile mutants derived from Δ*dps* mutant or wild-type *V*. *cholerae*. **(A) Distribution of *flrA*, *flrC*, *rpoN* mutation in nonmotile mutants derived from Δ*dps* mutant or wild-type *V*. *cholerae in vivo*.** The arrangement of the domains of FlrA, FlrC, RpoN were generated by Pfam database. The FlrA protein has three domains, an N-terminal flagellar regulatory protein FleQ domain, a central sigma-54 interaction domain and a C-terminal DNA binding helix turn helix (HTH) domain. The FlrC protein has three domains, an N-terminal response regulator receiver domain, a central sigma-54 interaction domain and a C-terminal DNA binding HTH domain. The RpoN protein has three domains, an N-terminal sigma-54 factor Activator interacting domain (AID), a central sigma-54 factor core binding domain (CBD) and a C-terminal DNA binding domain (DBD). Positions of the mutations in the domain were indicated with amino acid or nucleotide. The positions of mutations in nonmotile mutants derived from C6706 were showed by black, and derived from Δ*dps* were showed by blue. Red box represents identical mutation sites in nonmotile mutants derived from C6706 and Δ*dps*, * and number represents the number of mutants. **(B) Adult mice competition assay of *flrA*, *flrC*, *rpoN* point mutants.** We constructed point mutants of *flrA*, *flrC*, and *rpoN*, 10^8^ cells of wild-type and mutant were mixed in a 1:1 ratio and intragastrically administered to CD-1 adult mice. Fecal pellets were collected from each mouse at the fifth day post-infection, and plated onto selective plates. The competitive index (CI) was calculated as the ratio of mutant to wild-type colonies normalized to the input ratio. Horizontal line: median CI.

To investigate the mutation characteristics of the *flrA*, *flrC*, and *rpoN* genes, we mapped all the sequences of *flrA*, *flrC*, and *rpoN* of the nonmotile mutants in which motility was restored by the corresponding plasmids, either the original from C6706 (Fig 5A, black) or Δ*dps* (Fig 5A, blue), by Sanger sequencing (mutation sites were showed in S3 and S4 Table). The sequencing results showed that the types of mutations included deletions, insertions, duplications and base substitutions. Within the limited sample, we also found two identical mutation sites in nonmotile mutants derived from Δ*dps* or the wild-type (Fig 5A, red box), suggesting a similar mutation profile.

We further investigated whether these *flrA*, *flrC* and *rpoN* point mutations also affected *V*. *cholerae* colonization. Point mutations in *flrA*, *flrC* and *rpoN* were constructed in the corresponding knockout mutants by the introduction of spontaneous mutation sequences. In the competition assay with an isogenic wild-type strain, we found that all the point mutants of *flrA*, *flrC*, and *rpoN* also enhanced the ability of the bacteria to colonize the adult mouse intestine (Fig 5B). These results showed that the spontaneous mutations in the flagellar regulatory genes were derived from the Δ*dps* mutant or wild-type *V*. *cholerae in vivo*, probably through the same mechanism.

### Nonmotility-related mutations increase *V*. *cholerae* transmission between hosts

**Fig 6:**
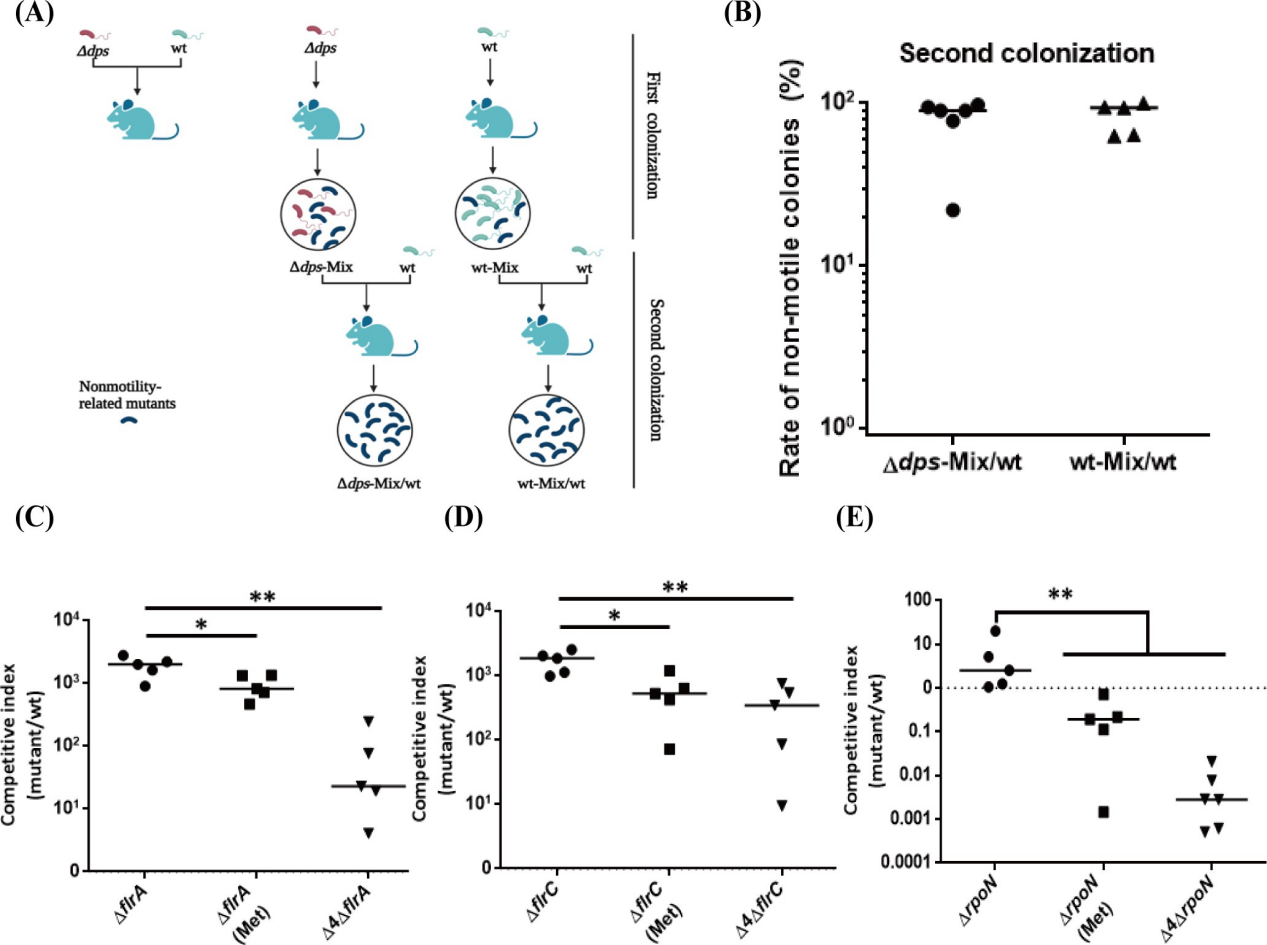
Nonmotility-related mutations increase *V*. *cholerae* transmission between hosts. **(A) Schematic experimental overview.** The illustration is created with BioRender.com. **(B) Rate of nonmotile mutants from second colonization in adult mice intestine.** Collection of *V*. *cholerae* from the feces of mice gavaged with Δ*dps* or wild-type alone on the fifth day of gavage (as Δ*dps-*Mix, wt-Mix), and then performed the competition colonization assay (second colonization) using wild-type C6706 and Δ*dps-*Mix or wt-Mix. Fecal pellets were collected at the fifth day post-infection, and plated on selective plates. One hundred *V*. *cholerae* colonies from one mouse were randomly selected for motility screening in 0.3% agar LB plates. Rate of nonmotile colonies were calculated as the ratio of nonmotile mutant colonies to all colonies per sample. Horizontal line: median. **Adult mice competition assay of methionine addition and Δ4Δ*flrA* (C), Δ4Δ*flrC* (D), Δ4Δ*rpoN* (E).** 10^8^ cells of wild-type and mutant were mixed in a 1:1 ratio and intragastrically administered to CD-1 adult mice. Methionine addition indicates adult mice supplemented with 25 mM L-methionine (Met) in drinking water. Fecal pellets were collected from each mouse at the fifth day post-infection, and plated onto selective plates. The competitive index (CI) was calculated as the ratio of mutant to wild-type colonies normalized to the input ratio. Δ4: Δ*metR*Δ*metI*Δ*metT*Δ*msrC*. Horizontal line: median CI. Significance was determined by Mann Whitney test, *p*-value: *, < 0.05, **, < 0.01.

We proved that the spontaneously generated nonmotile mutants had a colonization advantage in an adult mouse long-term colonization model and then further explored the possible physiological significance in the host. Competition colonization assay using wild-type C6706 and **Δ***dps* in an adult mouse model was performed, as shown in Fig 6A. We found that Δ*dps* was outcompeted by the wild-type in this model (S7A Fig, left), which is not surprising since Dps is a ROS response protein responsible for ROS resistance in the host [21]. However, bacteria recovered from mice infected with the Δ*dps* mutant only (Δ*dps-*Mix) outcompeted wild-type C6706 by approximately 100–1,000-fold (S7A Fig, middle). Similar results were observed when using bacteria from long-term colonized wild-type C6706 (wt-Mix) as inoculum (S7A Fig, right). The nonmotile rates of Δ*dps*-Mix/wt and wt-Mix/wt collected from the adult mice after two rounds of infection were almost 100% (Fig 6B). The results showed that *V*. *cholerae* cells obtained from the host gut had enhanced colonization ability due to the production of nonmotility-related mutations. Previous studies have shown that host colonization creates a hyperinfectious bacterial state, in which subsequent *V*. *cholerae* colonization is enhanced [36]. Here, our results showed that *V*. *cholerae* accumulated almost 100% of the nonmotility-related mutations after second colonization in adult mice, which may present a specific mechanism for the development of a hyperinfectious bacterial state during *V*. *cholerae* transmission between hosts.

To explore the mechanism of nonmotility-related mutations for enhanced colonization in adult mice. We performed the proteomic analysis between Δ*flrA* and Δ*flaA*, which represented the increased (Δ*flrA)* and decreased (Δ*flaA)* colonization in adult mice. The up-regulation of proteolysis pathway was confirmed at the translational level in Δ*flrA* by Gene Ontology (GO) enrichment analysis (S7B and S7C Fig). Methionine, one of the protein hydrolysis products, is thought to be associated with *V*. *cholerae* colonization in *Drosophila melanogaster* [37]. We hypothesized that methionine may be associated with *V*. *cholerae* colonization in adult mice, and performed the competition colonization assay in adult mice supplemented with 25 mM L-methionine (Met) in drinking water. The results showed Met supplementation reduced the ability of Δ*flrA*, Δ*flrC* and Δ*rpoN* to colonize adult mice (Fig 6C, 6D and 6E) by enhancement of absolute colony numbers of wild-type C6706 within intestine (S7D, S7E and S7F Fig). We further investigated whether methionine metabolism-related genes also affected Δ*flrA*, Δ*flrC* and Δ*rpoN* colonization. Based on published literature, we constructed Δ*metR*Δ*metI*Δ*metT*Δ*msrC* (Δ4) in Δ*flrA*, Δ*flrC* and Δ*rpoN* mutant and performed the competition colonization assay in adult mice, which showed the decreased advantage of colonization in adult mice (Fig 6C, 6D and 6E). These results suggested that methionine metabolism pathway may be one of the mechanism for Δ*flrA*, Δ*flrC* and Δ*rpoN* mutant increased colonization in adult mice.

**Fig 7:**
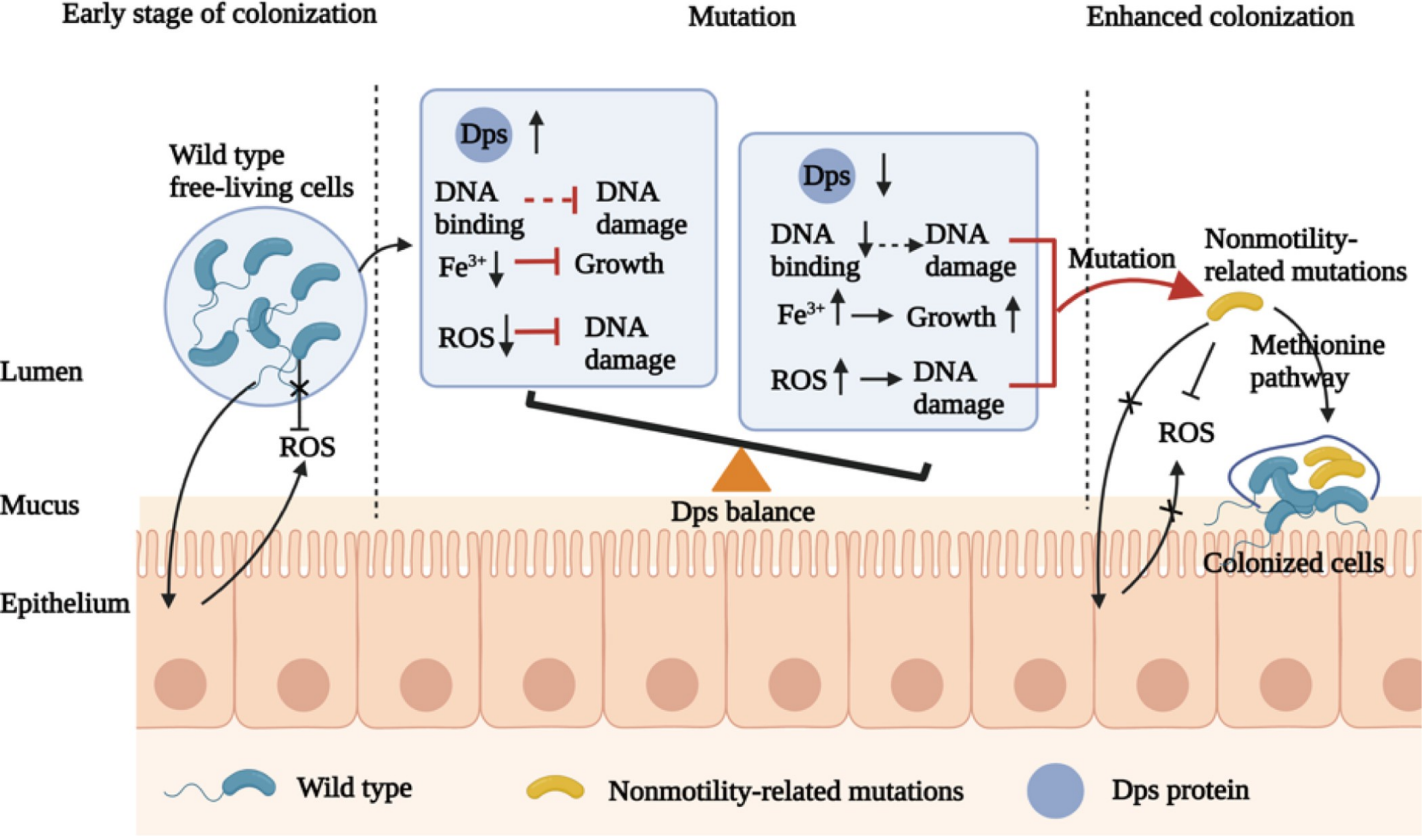
Proposed Model of generating nonmotility-related mutants. *V. cholerae* entering the mouse intestine are exposed to ROS, and the flagella of *V. cholerae* itself can also trigger inflammation. Dps is an important oxidative stress resistance protein, and its expression is significantly induced by ROS exposure. Increased Dps expression can confer resistance to ROS, but overexpression of Dps can also lead to sequestration of iron, inactivation of iron ion-dependent enzymes and inhibition bacterial growth. When the amount of Dps decreases, bacteria have decreased ability to resist ROS and DNA damage. *V. cholerae* produced nonmotility-related mutations, which had a stronger ability to resist ROS and increased fitness in the host by the methionine metabolism pathway. The illustration is created with BioRender.com.

In summary, *V*. *cholerae* entering the mouse intestine are exposed to ROS, and the flagella of *V*. *cholerae* itself can also trigger inflammation [38]. Dps is an important oxidative stress resistance protein, and its expression is significantly induced by ROS exposure [21]. However, the expression of the cycling Dps is highly dependent upon the growth phase, exhibiting downregulation at the exponential phase and upregulation at the starvation phase, and constitutive expression of *dps* hampers the growth of *V*. *cholerae* [24]. Increased Dps expression can confer resistance to oxidative stress, but overexpression can also lead to sequestration of iron, inactivation of iron ion-dependent enzymes and inhibition bacterial growth, which means that the Dps response to oxidative stress has a careful balance [39]. When the amount of Dps decreases, bacteria have decreased ability to resist ROS and DNA damage. *V*. *cholerae* produced nonmotility-related mutations, which had a stronger ability to resist ROS (S8A Fig) and increased fitness in the host. Nonmotility-related mutations fitness in the host may be via the methionine metabolism pathway (Fig 7).

## Discussion

The findings reported here reveal an evolutionary mechanism for *V*. *cholerae* adaptation to vertebrate hosts, which enhanced colonization in the adult mouse intestine via the spontaneous production of nonmotile mutants. The molecular mechanism of nonmotile mutants’ production is related to the deficiency of ROS detoxification function of the Dps protein. It appears that *V*. *cholerae* has evolved an efficient mechanism for high-frequency generation of nonmotile mutants that might provide an adaptive advantage under high levels of ROS in the host intestinal environment. Our findings are important to human public health, as they might explain why clinical isolates are nonmotile in cholera outbreak areas and facilitate the interpretation of epidemic strain genetic variation in future epidemics.

To date, several animal models have been developed to closely mimic Cholera to study the pathogenesis of *V*. *cholerae*. The infant mice model has enabled limited throughput studies of within-host virulence regulation, and oral gavage of *V*. *cholerae* leads to TCP (toxin coregulated pili, TCP)-dependent SI colonization that is highly similar to *V*. *cholerae* colonization in human volunteers [40,41]. Adult mice have become the model of choice for studies of adaptive immunity and the long-term colonization of *V*. *cholerae* [22,42]. *V*. *cholerae in vivo*-activated gene expression is distinct during infant mouse and adult mouse colonization. For example, the deletion of *dps* could colonize infant mice as well as wild-type but was defective for colonization in adult mice [21]. The mismatch repair system gene *mutS* knockout attenuated the ability of the bacteria to colonize infant mice, while the Δ*mutS* mutant outcompeted the wild-type in the later stages of infection in adult mice [28,43]. The *V*. *cholerae* TCP is critical for virulence, and deletion of *tcpA*, the major subunit of TCP, attenuated *V*. *cholerae* colonization in infant mice [41]. However, adult mouse colonization does not depend on the production of TCP [42]. Previous studies have reported that *flrA* is repressed and Δ*flrA*, Δ*flrC*, Δ*rpoN* are defective for colonization in infant mice [8,44]. We found that spontaneous mutations in the flagellar regulatory genes *flrA*, *flrC* and *rpoN* were strongly selected for during colonization, and the mutants exhibited a colonization advantage in adult mice. The results may represent a new pathogenesis mechanism of *V*. *cholerae* in response to selection stress in the gut.

Surprisingly, the obtained Δ*mutS* colonies did not contain a larger nonmotile population after 5 days of colonization in the adult mouse intestine (Fig 3D). In our previous study [43], Δ*mutS* enhanced the mutation rate for *V*. *cholerae in vivo* and *in vitro*, as shown with a rifampicin resistance assay (further confirmed in S3C and S3E Fig). A possible reason for this is that deletion of *mutS* leads to hypermutation in *V*. *cholerae*, so a large number of mutants derived from the *mutS* strain are produced and selected in adult mice by diverse adaptation advantages, of which the nonmotile trait is one, and the nonmotile mutants are not efficiently enriched. These results also indicated that spontaneous flagellar mutations during long-term colonization by wild-type *V*. cholerae in the host may represent a very likely direction for *V*. *cholerae* evolution.

A previous study showed that nonsynonymous SNPs (nsSNPs) mutations in conserved regions of FlrA were detected during long-term coincubation with *Acanthamoeba castellanii* (*A*. *castellanii*), and during long-term intra-amoebal host adaptation resulted in enhanced colonization of *V. cholerae* in zebrafish [45]. All of the nsSNPs of FlrA occurred in the central domain and one deletion occurred in the flanking region of the central domain and C-terminal HTH domain. Our results showed that the major mutation also occurred in the central domain of FlrA, while the mutation types were more diverse, including insertions, deletions and base substitutions, and the mutations covered the N-terminal, central and C-terminal domains of FlrA. The production of pyomelanin by *V*. *cholerae* confers resistance to predation by *A*. *castellanii*. A pyomelanin-overproducing mutant produces more ROS, which may account for the increased resistance to predation [46]. We speculate that mutations in *flrA* of *V*. *cholerae* during long-term coincubation with *A*. *castellanii* might also be associated with the ROS stress within *A*. *castellanii*. The natural habitat of zebrafish in Asia broadly overlaps with areas of cholera endemicity, suggesting that zebrafish and *V*. *cholerae* evolved in close contact with each other in the wild [47]. Our results showed that *flrA*, as a hotspot gene of nonmotile mutants, increases the potential for transmission and dissemination in the environment.

The competitive advantage of Δ*flrA* and Δ*flrC* is different from that of Δ*rpoN* in adult mice (Fig 2D). Although FlrA and FlrC are RpoN-dependent transcriptional regulators, deletion of *rpoN* does not completely silence *flrA* and *flrC* genes. The alternative σ factor (RpoN, σ^54^) of RNA polymerase recognizes gene promoter regions and initiates transcription. RpoN control the expression of many genes that might affect bacterial colonization and finally “hedge” against around 2-fold colonization advantage, which may be the reason why Δ*rpoN* colonization advantage is different from those of Δ*flrA* and Δ*flrC*. The colonization advantage of complemented strains Δ*flrA* (P_*lac*_-*flrA*), Δ*flrC* (P_*lac*_-*flrC*) and Δ*rpoN* (P_*lac*_-*rpoN*) in adult mice are different, just as the colonization advantage of the Δ*flrA*, Δ*flrC* and Δ*rpoN* knockout mutants. At chromosomal *lacZ* locus, we generated complemented strain that constitutively expresses complemented gene controlled by a constitutive P_*lac*_ promoter. It’s not surprising that the expression level of complemented gene controlled by a constitutive P_*lac*_ promoter is different from that of the wild-type gene.

We performed competition colonization assays with Δ*flrA*/wt, Δ*flrC*/wt, and Δ*rpoN*/wt in adult mice treated with 1% NAC to remove intestinal ROS. The results showed a decrease in the ability of Δ*flrA*, Δ*flrC* and Δ*rpoN* to colonize adult mice treated with NAC; however, the effect was not statistically significant for Δ*flrA* and Δ*flrC* (S8B Fig) and the mechanism by which the Δ*flrA*, Δ*flrC* and Δ*rpoN* mutations enhanced colonization in adult mice was different. This result suggested that nonmotile mutants (*flrA* and *flrC* mutation) had a stronger ability to resist ROS (S8A Fig), which may not be the main reason for the enhancement of colonization. Not surprisingly, Δ*flaA* attenuated *V*. *cholerae* colonization in adult mice (S2A Fig) but had a stronger ability to resist ROS (S8A Fig). We speculated that the *flrA*, *flrC*, and *rpoN* mutations increased the resistance to oxidative stress, which may be related to biofilm formation. It has been reported that the absence of the flagellar structure constitutes a signal to increase exopolysaccharide synthesis, which is an essential step for biofilm formation [48]. Alterations in different components of the flagellum influence the c-di-GMP-signaling modules that promote biofilm formation against oxidative stress [43,49]. Deletion of *vpsA* drastic reduction *Vibrio* polysaccharide (VPS) and biofilm production [50], we constructed Δ*vpsA* in Δ*flrA* and Δ*flrC* background and found biofilm is not responsible for enhanced colonization of Δ*flrA* and Δ*flrC* (S8C Fig). We have not constructed Δ*vpsA* in Δ*rpoN* due to deletion of *rpoN* significantly reduced the expression of genes in *vps* clusters [51]. In summary, spontaneous high-frequency mutations in the flagellar regulatory genes *flrA*, *flrC* and *rpoN* cause a nonmotile phenotype and enhance *V*. *cholerae* colonization in adult mice. The colonization advantage is independent of the motility and growth advantage of nonmotile mutants, ROS resistance and biofilm of *V*. *cholerae*. Further research is needed to elucidate the mechanism by which selected flagellar genes have a colonization advantage in adult mice.

To test whether the Dps binding function is related to nonmotile mutants, we first predicted the critical amino acid sites of Dps related to DNA binding function, and verified the function of the Dps mutant binding to DNA. Three positively charged lysine residues (K5, K8, and K10) in the N-terminal Dps tail were recognized as residues interacting with the negatively charged DNA backbone in *E*. *coli* [32]. The N-terminal of *V*. *cholerae* Dps has only one positively charged lysine residue (K15), unlike that of *E*. *coli* (S4 Fig). EMSA showed that the K15A mutant Dps protein could bind to the supercoiled plasmid pUC19 and produce larger protein-DNA complexes that migrate slower than the free form of pUC19 (S9 Fig). The other DNA binding signature that has been identified is the C-terminal region of *M*. *smegmatis* Dps; removal of the 16 C-terminal residues of Dps, containing five positively charged amino acids, did not show DNA binding activity [52]. The C-terminal of *V*. *cholerae* Dps has three positively charged amino acid residues (R142, K146, K156); however, both the R142AK146AK156A mutant Dps and DpsΔ16C could bind to the supercoiled plasmid pUC19 (S9 Fig). We predicted 7 positively charged surface amino acid residues (K156, K44, K87, K92, H80, R77, K146) in Dps candidates for binding to DNA by NetSurfP-2.0 [53] and created K44A, K87A, K92A, H80A, R77A Dps mutants. The K156A and K146A mutants were not to be constructed because the R142AK146AK156A mutant Dps could bind to the supercoiled plasmid pUC19. The EMSA results showed that all mutant Dps proteins could bind to the supercoiled plasmid pUC19 (S9 Fig). We failed to construct a *V*. *cholerae* Dps protein without DNA binding activity. It was reported that Dps binding sites across the bacterial chromosome are nonrandomly distributed and prone to enrichment in inverted repeats [54]. Further research is needed to determine whether the Dps binding site preference is related to the mutations in flagellar genes.

In summary, in the present study, we presented evidence that Dps ROS detoxification function deficiency in the adult mouse intestine results in a high frequency of spontaneous mutations in flagellar regulatory genes, which enhances colonization in adult mice. These phenotypic and genotypic changes help us to understand the potential factors responsible for mutants of cholera epidemics in cholera-endemic countries.

## Materials and methods

### Ethics statement

The animal experiments were performed with protocols approved by the Ethical Committee of Huazhong University of Science and Technology (Permit Number: SYXK (E) 2016–0057).

### Bacterial strains, plasmids and culture conditions

The strains and plasmids used in this study are listed in S1 Table. *V*. *cholerae* EI Tor biotype C6706 [55] was used as a parental strain. The in-frame deletions were constructed by a previously described method [56,57], the upstream and downstream flanking DNA fragments were amplified and cloned into the suicide vector pWM91 for subsequent *sacB*-mediated allelic exchange in *V*. *cholerae*. The *flrA*, *flrC*, and *rpoN* point mutants were constructed in knockout mutants by the introduction of spontaneous mutation sequences containing *flrA*1384_1385dupG, *flrA*389delA, *flrC*720_722delAGC, *flrC*370_388delAAGTCAGATGACAACGGCG, and *rpoN*762_763insA. The *dps* point mutants were constructed in the knockout mutant by introduction of a *dps* knock-in sequence containing *dps*^D65A^ or *dps*^F46E^. All mutants were confirmed by DNA sequencing.

*V*. *cholerae* and *E*. *coli* were grown at 37°C in Luria-Bertani (LB) medium or LB medium supplemented with antibiotics at the following concentrations: streptomycin (100 μg/ml), ampicillin (100 μg/ml), kanamycin (50 μg/ml), rifampicin (50 μg/ml) and chloramphenicol (2 μg/ml for *V*. *cholerae* and 25 μg/ml for *E*. *coli*).

### Electron microscopy

*V*. *cholerae* were grown to mid-log phase in LB, and then suspended in 0.9% NaCl. The samples were loaded on a carbon-coated grid and stained with 1% phosphotungstic acid before electron microscopy (HT7700, Japan).

### Collection of nonmotile *V*. *cholera* mutants from mouse faeces

For the antibiotic cocktail-treated mouse model, five-week-old female CD-1 mice were treated with an antibiotic cocktail in drinking water containing a final concentration of 0.4 mg/mL kanamycin, 0.035 mg/mL gentamycin, 850 U/mL colistin, 0.215 mg/mL metronidazole, 0.045 mg/mL vancomycin, 0.5 mg/mL cefoperazone, and 0.2 mg/mL aspartame for 3 days. Then, 10 g/L streptomycin was added to the drinking water for the remainder of the experiment.

For the NAC-treated mouse model, NAC was used as a scavenger of reactive oxygen species [35], and five-week-old CD-1 mice were treated with drinking water containing 1% NAC (Sigma) for 7 days. Then, 10 g/L streptomycin was added to the 1% NAC drinking water for the remainder of the experiment.

One day after streptomycin treatment, approximately 10^8^ CFU of *V*. *cholerae* were intragastrically inoculated into each mouse. To avoid a cage effect during mutant collection, we used a single-cage single-mouse infection experiment to ensure that the *V*. *cholerae* isolates recovered from each mouse were derived from a single mutation event. Fecal pellets were collected from each mouse at 5 days post-infection and homogenized in 1.5 ml of LB medium. Serial dilutions were plated on plates containing streptomycin. One hundred *V*. *cholerae* colonies from one mouse were randomly selected for motility screening in 0.3% agar LB plates, and we used 0.99 instead of 0 to calculate the rate of nonmotility. To avoid picking siblings of the same bacterium, we picked only 3–4 nonmotile colonies per mouse for the next experiment.

### Bacterial DNA extraction and sequencing

To avoid picking siblings of the same bacterium, we picked only 3–4 nonmotile colonies per mouse. A total of 53 *V*. *cholerae* nonmotile mutants from 16 mice were grown separately in LB medium to logarithmic phase and mixed 1:1 to extract DNA. Three technical repeats named AC1, AC2, and AC3 were used for subsequent sequencing experiments to determine the mutations in the genome. The sequencing reads were subjected to quality control using fastp v0.20.1 [58]. High quality sequences were used for genomic alignments based on the *V*. *cholerae* reference genomes NC_002505.1 and NC_002506.1 by bwa-0.7.17 [59]. SnpEff 4.3 was used for the annotation and functional analysis of SNPs [27]. The SnpEff impact category HIGH was used to filter SNPs for putative High impact variants (large chromosome deletion, exon deletion, insertion/deletion frame shift, donor splice site disruptions, acceptor splice site disruptions, stop codon gains, stop codon losses, start losses) [60]. Sequencing data are deposited in NCBI’s Sequence Read Archive (SRA) under the project accession number PRJNA831328. All High impact variants data are included in the additional files in S2 Table.

### Adult mouse competition assay

The streptomycin-treated adult mouse model was used to examine *V*. *cholerae* colonization as previously described [22] with the following modifications. Two days after 10 g/L streptomycin drinking water treatment, approximately 10^8^ C6706 (*lacZ*-) and mutant (*lacZ*+) were mixed together at a 1:1 ratio and inoculated intragastrically into 5-week-old female CD-1 mice. This streptomycin drinking water treatment was maintained throughout this experiment. Two or three fecal pellets were collected from each mouse on day 5 after inoculation, suspended in LB medium, serially diluted, and then plated on plates containing 5-bromo-4-chloro-3-indo-lyl-β-D-galactopyranoside (X-gal) and streptomycin. The competitive index was calculated as the ratio of mutant to wild-type colonies normalized to the input ratio.

### Electrophoretic mobility shift assays (EMSAs)

Wild-type and Dps mutant proteins without a hexahistidine tag were expressed and purified as previously described [34]. Electrophoretic mobility shift assays were performed as previously described [61]. Different concentrations of wild-type or Dps mutant protein were incubated with 0.6 pM supercoiled plasmid pUC19 (in 50 mM MOPS buffer (pH 7.0) containing 50 mM NaCl) for 10 min at 25°C. Electrophoresis was performed in 1% agarose gels in TAE buffer (40 mM Tris-acetate buffer pH 8.0, 0.1 mM EDTA) at room temperature for 1 h 30 min at 80 V. The gels were stained with SYBR Safe solution (Invitrogen) for 30 min and imaged.

### Mutation frequency assays

Overnight cultures of the wild-type, Δ*mutS*, and Δ*dps* were inoculated into fresh LB with or without 300 μM H_2_O_2_ and grown at 37°C with shaking for 12 h. The cultures were then plated on LB agar and LB agar with rifampicin (50 μg/ml). After overnight growth, the *in vitro* mutation frequency was scored as the number of rifampicin-resistant colonies. The *in vivo* mutation frequency was determined with the protocol previously described [43]. Fecal pellets from mice gavaged with *V*. *cholerae* were collected at the fifth day post-infection and homogenized in LB medium with streptomycin. After brief centrifugation, the supernatants were incubated at 37°C on a shaker for 12 h. The cultures were then serially diluted onto LB agar with streptomycin (500 μg/ml) and LB agar with rifampicin (50 μg/ml) and streptomycin (500 μg/ml). After overnight growth, rifampicin resistant colonies were scored.

### Fluorescence staining for ROS

Five-week-old female CD-1 mice were treated with antibiotic cocktail drinking water for 3 days or drinking water containing 1% NAC (Sigma) for 7 days. The small intestine tissue blocks were harvested at the time point and immediately submerged in liquid nitrogen. To detect ROS levels in small intestine tissue, ROS staining solution (Sigma) was added to the sample and incubated for 30 minutes at 37°C, and nuclei were stained with DAPI solution at room temperature for 10 min. The sample was washed with PBS. The fluorescence was detected with an excitation wavelength of 510–560 nm and emission wavelength of 590 nm for ROS and 330–380 nm excitation and 420 nm emission for DAPI by microscopy (Nikon Eclipse C1, Japan). The fluorescence densities of ROS were measured with ImageJ software.

### Proteomic analysis

Overnight cultures of Δ*flrA* and Δ*flaA* were 1:1,00 sub-cultured into fresh 50 mL LB medium and grown at 37°C, 200 rpm until *logarithmic growth* phase. Bacterial precipitate was collected and used for proteomic measurements as previously described [62]. Raw MS files were searched with the MaxQuant software (http://maxquant.org/, Version 1.5.3.30) against the *V*. *cholerae* N16961 protein database. Proteins with fold changes > 1.5 were further compiled and considered as candidates differing between samples. Candidate proteins were performed Gene Ontology (GO) enrichment analysis and grouped into the GO pathway by DAVID. The proteomic analysis data are included in the additional files in S5 Table.

## Supporting information

S1 Fig Competition growth of Δ*flrA*/wt, Δ*flrC*/wt and Δ*rpoN*/wt. The competition mixture of Δ*flrA*/wt **(A)**, Δ*flrC*/wt **(B)** and Δ*rpoN*/wt **(C)** were cultured in LB media in anaerobic tubes on 37°C to mimic *in vivo* experiments. We cultivated the competition mixture for 5 days and transfer the competition mixture into fresh media each day to mimic continuous availability of nutrients (transfer culture) or 5 days in the same media (continuous culture). The competitive index (CI) was calculated as the ratio of mutant to wild-type colonies normalized to the input ratio. Horizontal line: mean CI.
(TIF)

S2 Fig Adult mice competition assay of Δ*flaA*, Δ*tagE* and Δ*mshQ* mutant. 10^8^ cells of wild-type and Δ*flaA* **(A)**, Δ*tagE*, Δ*mshQ* mutant **(B)** were mixed respectively in a 1:1 ratio and intragastrically administered to CD-1 adult mouse, respectively. The competitive index (CI) of the fifth day after infection was calculated as the ratio of mutant to wild-type colonies normalized to the input ratio. Horizontal line: median CI.
(TIF)

S3 Fig Δ*dps* does not affect the *V*. *cholerae* motility and mutation rate. **(A) Rate of nonmotile mutants in Δ*dps* and complemented strains in adult mice intestine.** Δ*dps* and complemented strains were intragastrically inoculated individually into CD-1 mice treated with antibiotics cocktail. Fecal pellets were collected at the fifth day post-infection, and plated on selective plates. One hundred *V*. *cholerae* colonies from one mouse were randomly selected for motility screening in 0.3% agar LB plates. Rate of nonmotile colonies were calculated as the ratio of nonmotile mutant colonies to all colonies per sample. Horizontal line: median. Significance was determined by Mann Whitney test, *p*-value: *, < 0.05. **(B) Growth of wild-type and Δ*lexA* in M9 minimal medium.** Exponentially growing cultures of wild-type C6706 (blue) and Δ*lexA* (orange) were grown in M9 minimal medium (M9 salts plus 2 mM MgSO_4_, 0.1 mM CaCl_2_, and 0.2% glucose as the sole carbon source). The recovery and growth of each strains were monitored over time. The averages of 3 experiments were showed for each strain. (**C) Mutation rate of Δ*mutS*, Δ*nfo*, Δ*xthA* and Δ*uvrA in vivo*.** Fecal pellets from mice gavaged with Δ*mutS*, Δ*nfo*, Δ*xthA*, Δ*uvrA* alone were collected and homogenized in LB medium with streptomycin. After brief centrifugation, the supernatants were incubated at 37°C shaker for 12 h. The cultures were then serial diluted onto LB agar with streptomycin and LB agar with rifampicin and streptomycin. After overnight growth, rifampicin resistance colonies were scored. Horizontal line: median. Significance was determined by Mann Whitney test; *p*-value: ns, not significant, **, < 0.01. **(D) Motility phenotype of Δ*dps* mutant.** Bacteria were inoculated into 0.3% agar LB plates and incubated at 37°C for 8 h. C6706, motility positive control. Δ*flaA*, motility negative control. **(E) Mutation rate of wild-type, Δ*mutS* and Δ*dps in vitro*.** Overnight cultures of wild-type, Δ*mutS* and Δ*dps* were inoculated into fresh LB with or without H_2_O_2_ and grown at 37°C shaking for 12 h. The cultures were then plated on LB agar and LB agar with rifampicin. After overnight growth, rifampicin resistance colonies were scored. Horizontal line: average. Significance was determined by One-way ANOVA; *p*-value: ns, not significant, **, < 0.01, ***, < 0.001. (**F) Mutation rate of wild-type and Δ*dps in vivo*.** Horizontal line: median. Significance was determined by Mann Whitney test; *p*-value: ns, not significant.
(TIF)

S4 Fig Alignment of representative sequences of Dps proteins. The software Multalin was used to align Dps sequences. Sequence alignment of Dps and Dps-like proteins from the NCBI protein database. *Vibrio choleare* (Dps-*Vibrio*, WP_000224703), *Helicobacter pylori* (Dps-*Helicobacter*, WP_180632519), *Listeria innocua* (Dps-*Listeria*, SPX75031) *Escherichia coli* (Dps-*E*. *coli*, WP_000100800), *Shigella boydii* (Dps-*Shigella*, QQT73920), *Rahnella aquatilis* (Dps-*Rahnella*, WP_047608693), *Streptomyces albidoflavus* (Dps-*Streptomyces*, TWV28064), *Mycolicibacterium smegmatis* (Dps-*Mycolicibacteri*, VTP10463), *Corynebacterium glutamicum* (Dps-*Corynebacterium*, WP_211439578). High consensus residues were in red and low consensus residues in blue. The conserved residues at the ferroxidase site according to *E*. *coli* Dps were showed by *, and the conserved residue at the Dps to ferritin structural switch site according to *Mycolicibacterium smegmatis* Dps was shown in ^.
(TIF)

S5 Fig Detected of ROS level of small intestine tissue from mice treated with antibiotics cocktail or NAC. **(A) Fluorescent images of ROS level of small intestine tissue.** Mice were treated with antibiotics cocktail (ROS+) or NAC (ROS-). Small intestine tissue was harvested and intracellular ROS was labelled with ROS staining solution (red), and nuclei was stained with DAPI (blue). All images were collected under a microscope. Bars represent 50 μm. **(B) Mean fluorescence densities of ROS.** Independent-samples *t* test was used for data analysis. *p*-value: *, < 0.05.
(TIF)

S6 Fig Reparable motility phenotype of nonmotile mutants derived from Δ*dps*. The pBBR-P_*bad*_-*flrA*, pBBR-P_*bad*_-*flrC*, pBBR-P_*bad*_-*rpoN* plasmids were constructed to complement the motility phenotype of fifty-one nonmotile mutants derived from Δ*dps*. Reparable motility phenotype was detected by 0.3% agar LB plates and incubated at 37°C for 8 h.
(TIF)

S7 Fig Proteomic analysis of Δ*flrA* and Δ*flaA*. **(A) Adult mice competition assay of *V*. *cholerae* from mouse intestinal filtration.** We collection of *V*. *cholerae* from the feces of mice gavaged with Δ*dps* or wild-type C6706 alone at the fifth day post-infection (as Δ*dps*-Mix, wt-Mix), and then performed the competition colonization assay using wild-type C6706 and Δ*dps* (left), Δ*dps*-Mix (middle) or wt-Mix (right), the competitive index (CI) of the fifth day after infection was calculated as the ratio of mutant to wild-type colonies normalized to the input ratio. Horizontal line: median CI. Significance was determined by Kruskal-Wallis test, *p*-value: **, < 0.01. **Gene Ontology (GO) enrichment analysis of up-regulated pathways (B) and down-regulated pathways (C) of proteomic data.** We performed the bacterial precipitate proteomic analysis of Δ*flrA* and Δ*flaA*, which represented the increased (Δ*flrA)* and decreased (Δ*flaA)* colonization in adult mice. Up-regulated/Down-regulated pathways indicate increased/decreased expression in Δ*flrA*. See supplementary information for a complete list with proteomic data. **Absolute colony numbers of wild-type C6706 and Δ*flrA* (D), Δ*flrC* (E), Δ*rpoN* (F) from adult mice competition assay with or without methionine.** Methionine addition indicates adult mice supplemented with 25 mM L-methionine (Met) in drinking water. Horizontal line: median. Significance was determined by Mann Whitney test, *p*-value: *, < 0.05, **, < 0.01.
(TIF)

S8 Fig ROS resistance and VPS did not the main reasons for the nonmotility-related mutations enhanced colonization in adult mice. **(A) Growth of wild-type and Δ*flrA*, Δ*flrC*, Δ*rpoN* under ROS stress.** Exponentially growing cultures of wild-type C6706 (Blue), Δ*flrA* (red), Δ*flrC* (green), Δ*rpoN* (purple) and Δ*flaA* (orange) were grown in LB with 600 μM H_2_O_2_. The recovery and growth of each strains were monitored over time. The averages of 3 experiments were showed for each strain. **(B) Colonization of Δ*flrA*, Δ*flrC*, Δ*rpoN* mutants in adult mice treated with or without NAC.** 10^8^ cells of wild-type and mutant were mixed in a 1:1 ratio and intragastrically administered to CD-1 adult mice treated with or without NAC. The competitive index (CI) of the fifth day after infection was calculated as the ratio of mutant to wild-type colonies normalized to the input ratio. Horizontal line: median CI. Significance was determined by Mann Whitney test, *p*-value: ns, not significant, *, < 0.05. **(C) Adult mice competition assay of Δ*flrA*Δ*vpsA*, Δ*flrC*Δ*vpsA*.** Horizontal line: median CI. Significance was determined by Mann Whitney test, *p*-value: ns, not significant.
(TIF)

S9 Fig Binding of Dps to supercoiled plasmid pUC19. Different concentration K15A, R142AK146AK156A, Δ16C, K44A, R77A, H80A, K87A and K92A of Dps mutant protein was incubated individually with 0.6 pM of supercoiled plasmid pUC19 (in 50 mM MOPS buffer pH 7.0, containing 50 mM NaCl).
(TIF)

S1 Table Strains and plasmids used in this study. (DOCX)

S2 Table Distribution of mutations in nonmotility-related mutants. (XLSX)

S3 Table *flrA*, *flrC*, *rpoN* gene mutation sites in wild-type C6706 *in vivo*. (XLSX)

S4 Table *flrA*, *flrA*, *rpoN* gene mutation sites in *Δdps in vivo*. (XLSX)

S5 Table Proteomic analysis of Δ*flrA* and Δ*flaA*. (XLSX)
