# Supplementary figures and images for: Dps-dependent in vivo mutation enhances long-term host adaptation in Vibrio cholerae
Source: PLoS Pathog. 2023 Mar 16;19(3):e1011250. doi: 10.1371/journal.ppat.1011250 (PMC10104298; doi:10.1371/journal.ppat.1011250)

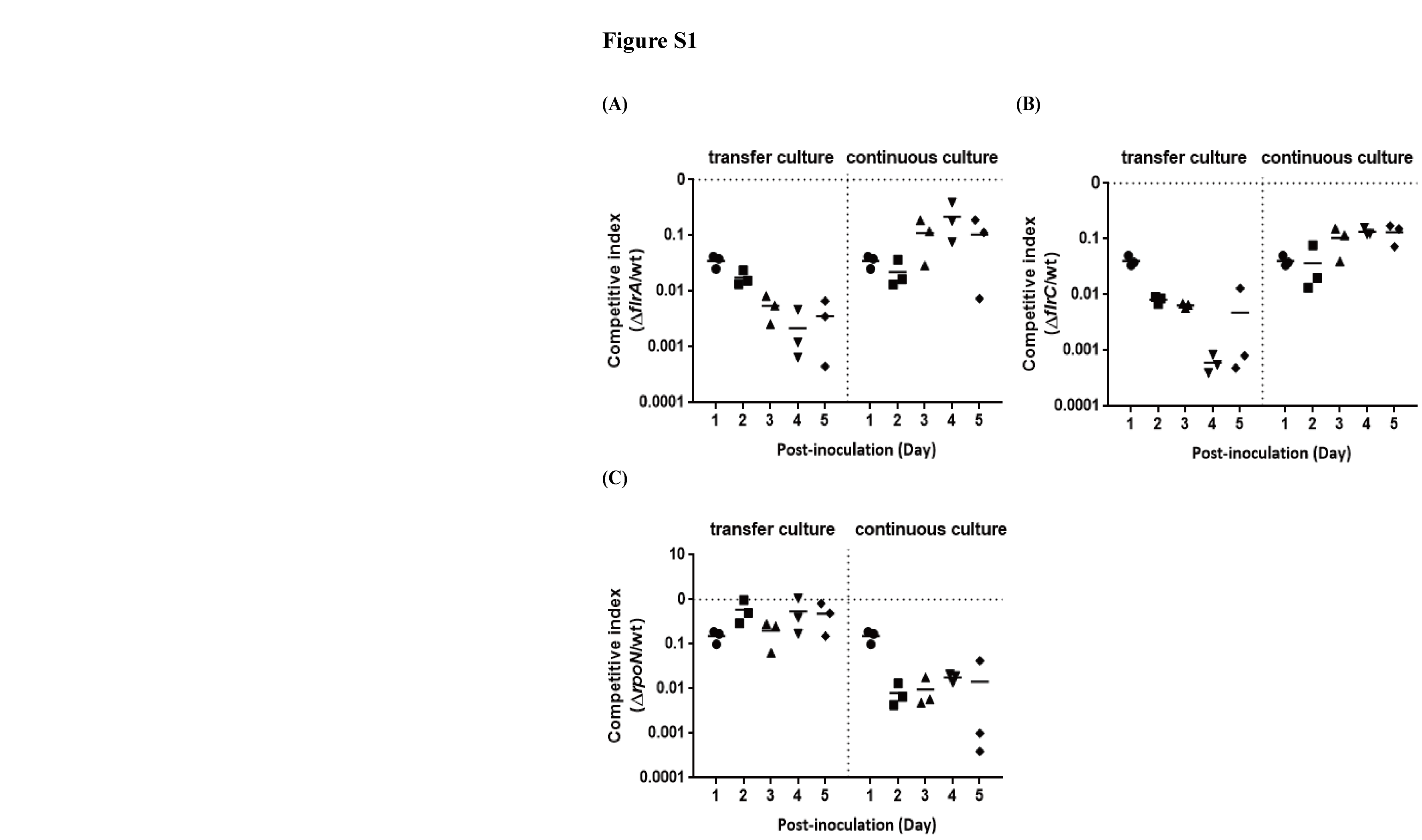

Supplement: S1 Fig — The competition mixture of ΔflrA/wt (A), ΔflrC/wt (B) and ΔrpoN/wt (C) were cultured in LB media in anaerobic tubes on 37°C to mimic in vivo experiments. We cultivated the competition mixture for 5 days and transfer the competition mixture into fresh media each day to mimic continuous availability of nutrients (transfer culture) or 5 days in the same media (continuous culture). The competitive index (CI) was calculated as the ratio of mutant to wild-type colonies normalized to the input ratio. Horizontal line: mean CI. (TIF) [file ppat.1011250.s001.tif]

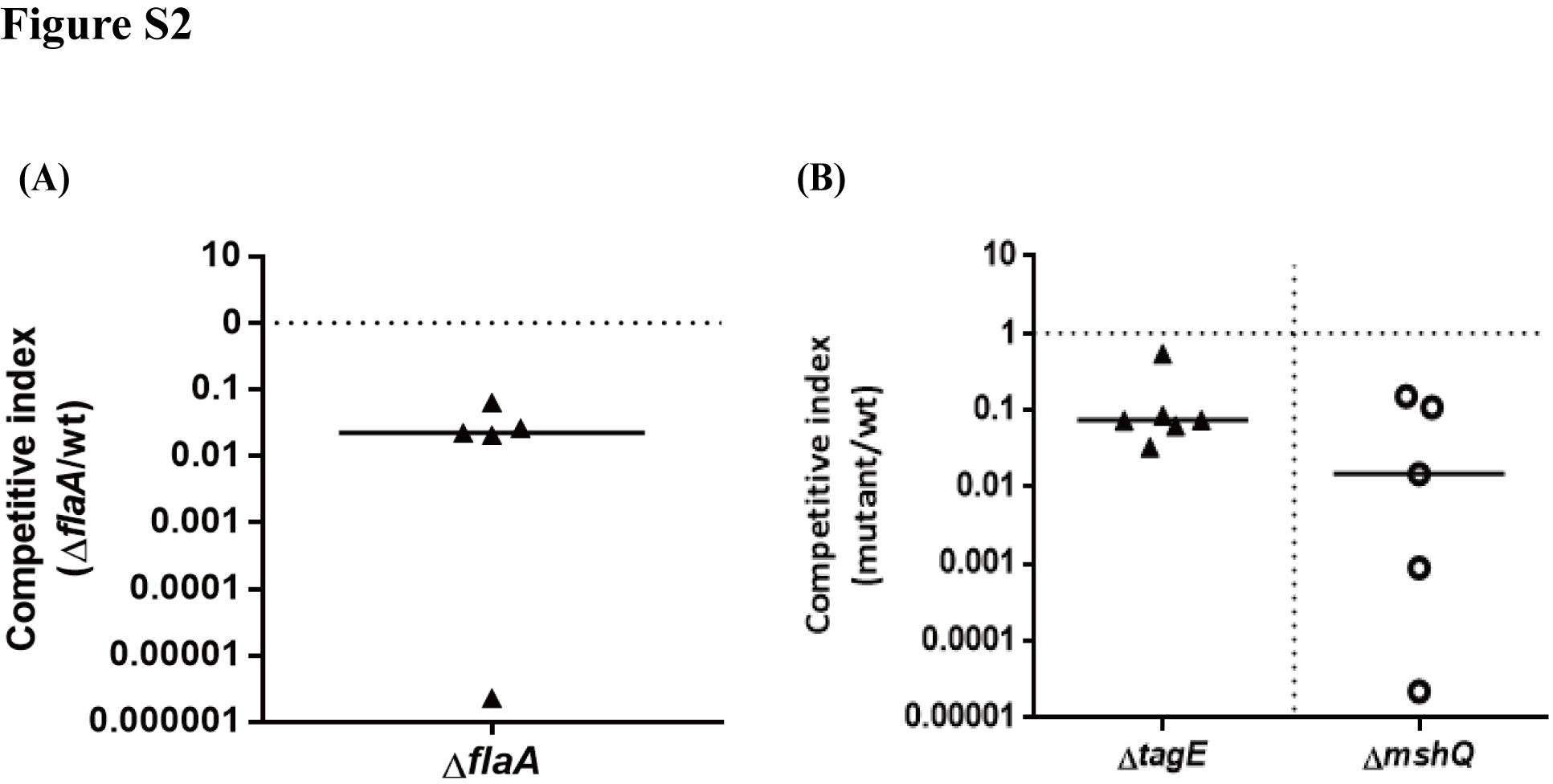

Supplement: S2 Fig — 108 cells of wild-type and ΔflaA (A), ΔtagE, ΔmshQ mutant (B) were mixed respectively in a 1:1 ratio and intragastrically administered to CD-1 adult mouse, respectively. The competitive index (CI) of the fifth day after infection was calculated as the ratio of mutant to wild-type colonies normalized to the input ratio. Horizontal line: median CI. (TIF) [file ppat.1011250.s002.tif]

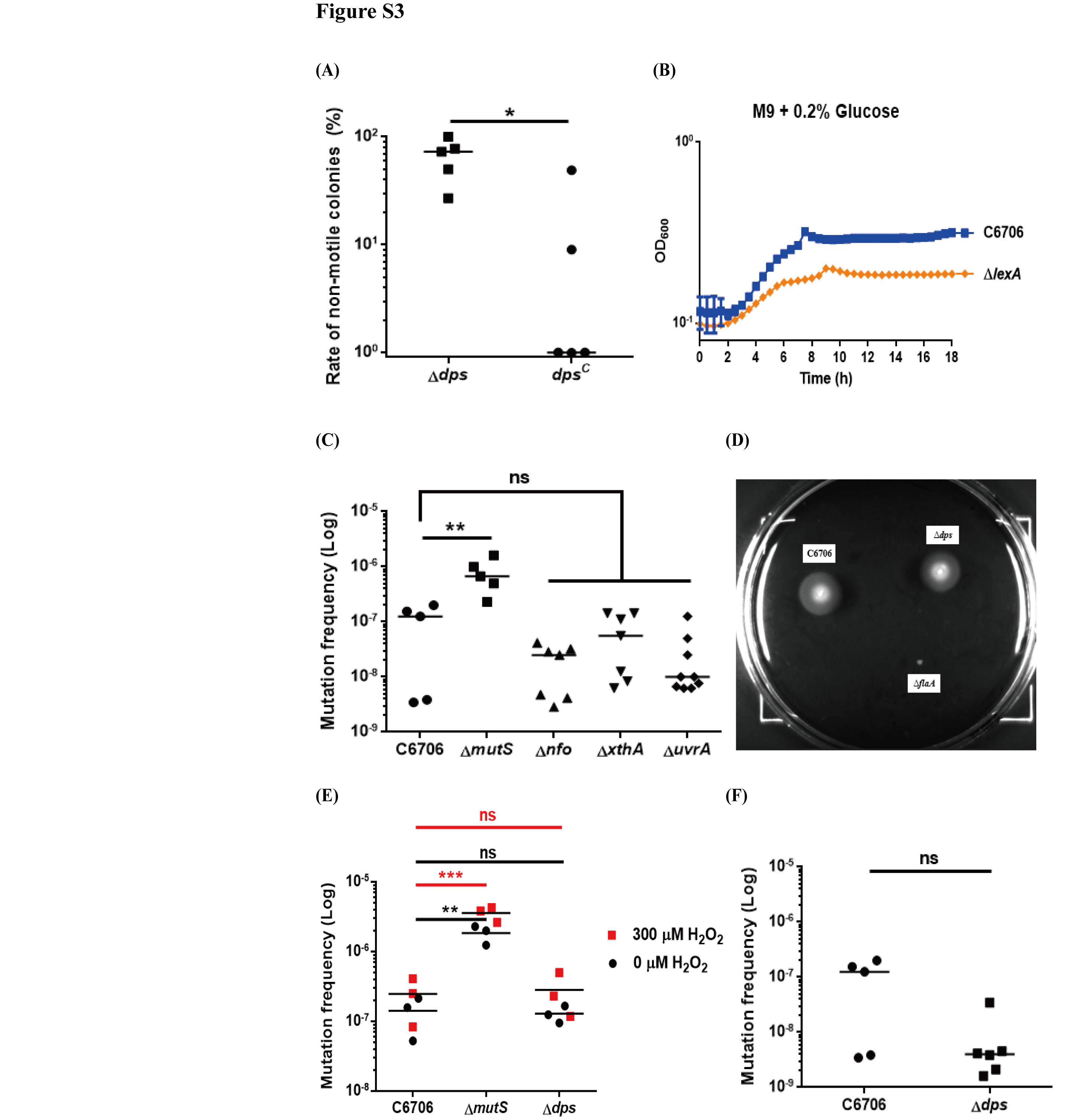

Supplement: S3 Fig — (A) Rate of nonmotile mutants in Δdps and complemented strains in adult mice intestine. Δdps and complemented strains were intragastrically inoculated individually into CD-1 mice treated with antibiotics cocktail. Fecal pellets were collected at the fifth day post-infection, and plated on selective plates. One hundred V. cholerae colonies from one mouse were randomly selected for motility screening in 0.3% agar LB plates. Rate of nonmotile colonies were calculated as the ratio of nonmotile mutant colonies to all colonies per sample. Horizontal line: median. Significance was determined by Mann Whitney test, p-value: *, < 0.05. (B) Growth of wild-type and ΔlexA in M9 minimal medium. Exponentially growing cultures of wild-type C6706 (blue) and ΔlexA (orange) were grown in M9 minimal medium (M9 salts plus 2 mM MgSO4, 0.1 mM CaCl2, and 0.2% glucose as the sole carbon source). The recovery and growth of each strains were monitored over time. The averages of 3 experiments were showed for each strain. (C) Mutation rate of ΔmutS, Δnfo, ΔxthA and ΔuvrA in vivo. Fecal pellets from mice gavaged with ΔmutS, Δnfo, ΔxthA, ΔuvrA alone were collected and homogenized in LB medium with streptomycin. After brief centrifugation, the supernatants were incubated at 37°C shaker for 12 h. The cultures were then serial diluted onto LB agar with streptomycin and LB agar with rifampicin and streptomycin. After overnight growth, rifampicin resistance colonies were scored. Horizontal line: median. Significance was determined by Mann Whitney test; p-value: ns, not significant, **, < 0.01. (D) Motility phenotype of Δdps mutant. Bacteria were inoculated into 0.3% agar LB plates and incubated at 37°C for 8 h. C6706, motility positive control. ΔflaA, motility negative control. (E) Mutation rate of wild-type, ΔmutS and Δdps in vitro. Overnight cultures of wild-type, ΔmutS and Δdps were inoculated into fresh LB with or without H2O2 and grown at 37°C shaking for 12 h. The cultures were then plated on LB [file ppat.1011250.s003.tif]

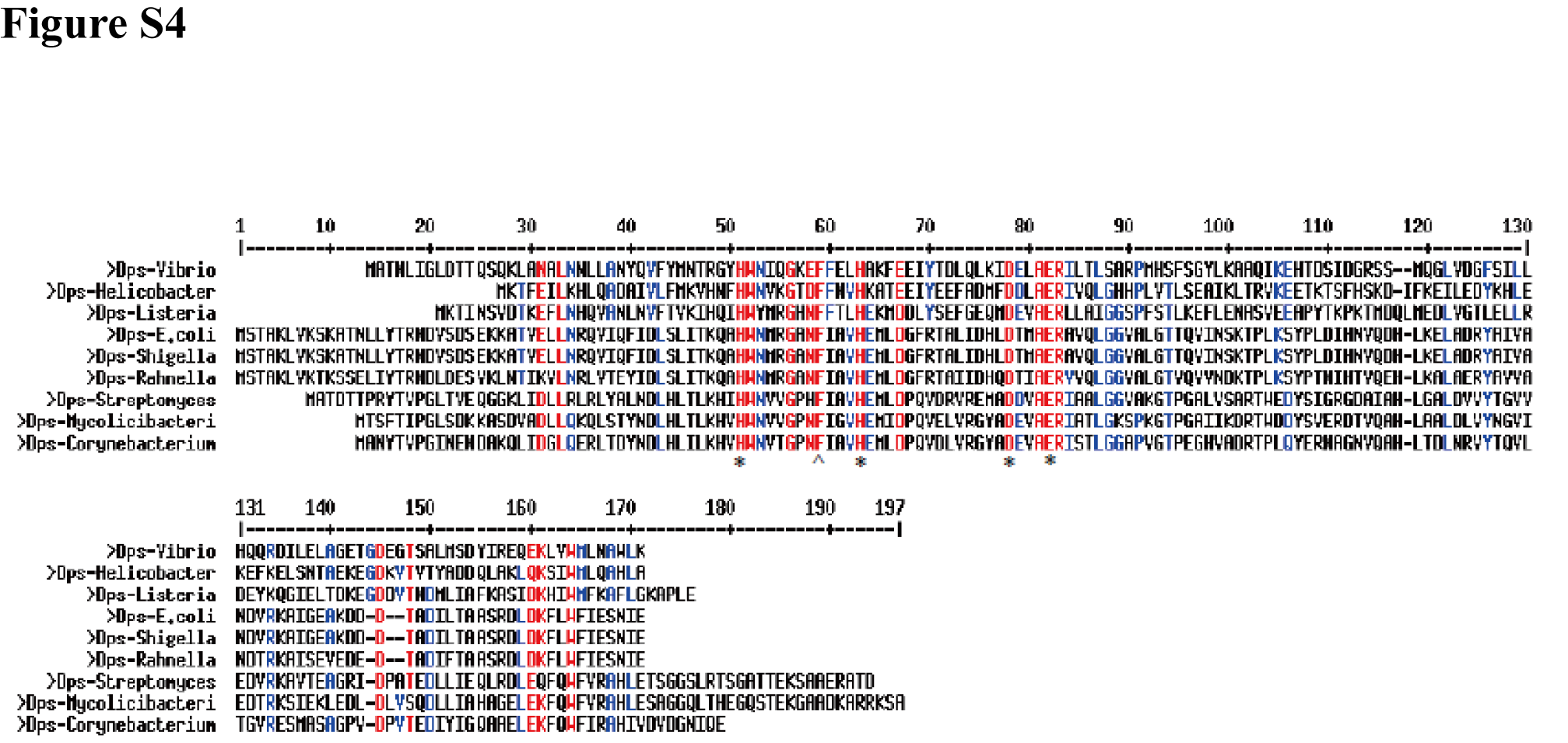

Supplement: S4 Fig — The software Multalin was used to align Dps sequences. Sequence alignment of Dps and Dps-like proteins from the NCBI protein database. Vibrio choleare (Dps-Vibrio, WP_000224703), Helicobacter pylori (Dps-Helicobacter, WP_180632519), Listeria innocua (Dps-Listeria, SPX75031) Escherichia coli (Dps-E. coli, WP_000100800), Shigella boydii (Dps-Shigella, QQT73920), Rahnella aquatilis (Dps-Rahnella, WP_047608693), Streptomyces albidoflavus (Dps-Streptomyces, TWV28064), Mycolicibacterium smegmatis (Dps-Mycolicibacteri, VTP10463), Corynebacterium glutamicum (Dps-Corynebacterium, WP_211439578). High consensus residues were in red and low consensus residues in blue. The conserved residues at the ferroxidase site according to E. coli Dps were showed by *, and the conserved residue at the Dps to ferritin structural switch site according to Mycolicibacterium smegmatis Dps was shown in ^. (TIF) [file ppat.1011250.s004.tif]

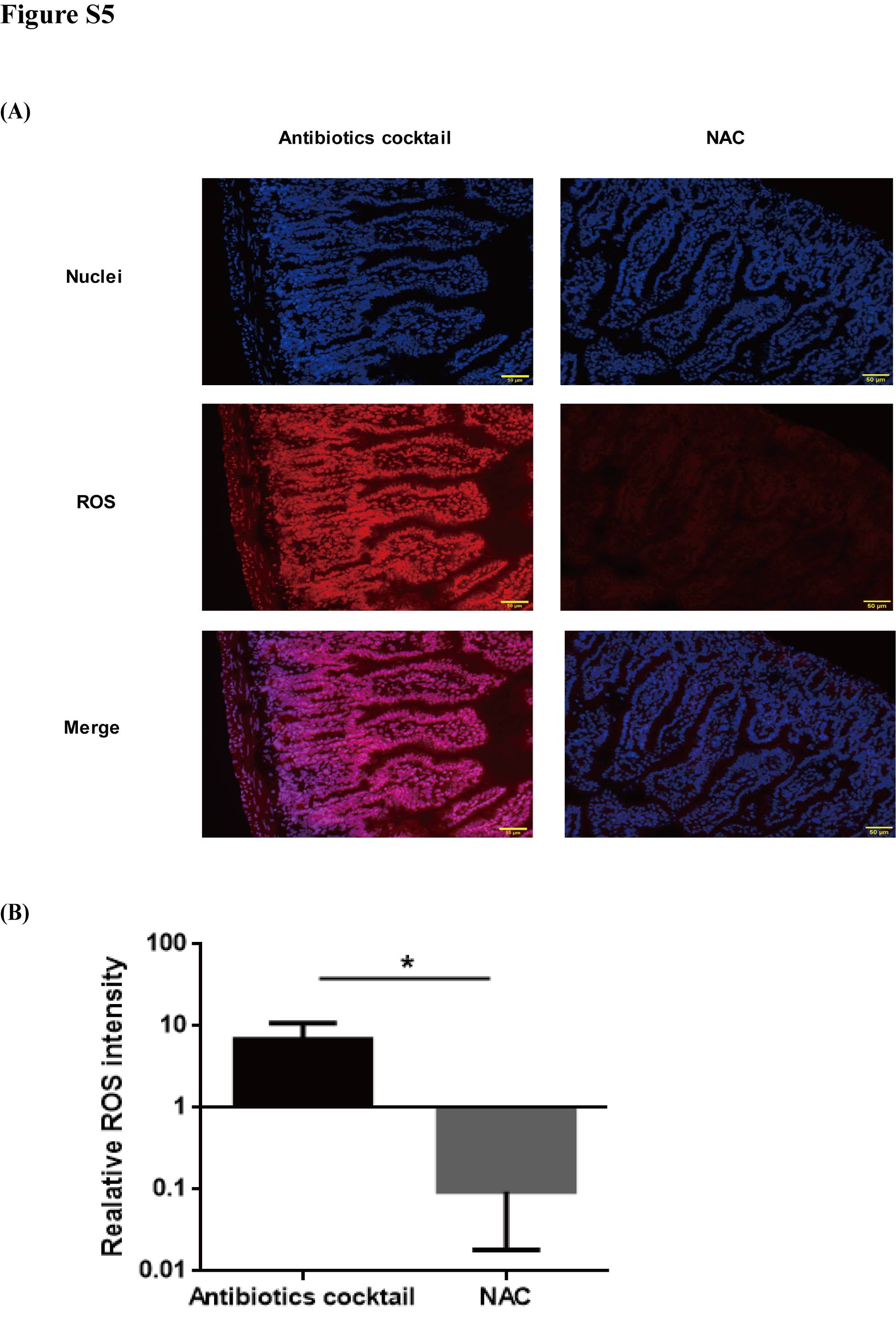

Supplement: S5 Fig — (A) Fluorescent images of ROS level of small intestine tissue. Mice were treated with antibiotics cocktail (ROS+) or NAC (ROS-). Small intestine tissue was harvested and intracellular ROS was labelled with ROS staining solution (red), and nuclei was stained with DAPI (blue). All images were collected under a microscope. Bars represent 50 μm. (B) Mean fluorescence densities of ROS. Independent-samples t test was used for data analysis. p-value: *, < 0.05. (TIF) [file ppat.1011250.s005.tif]

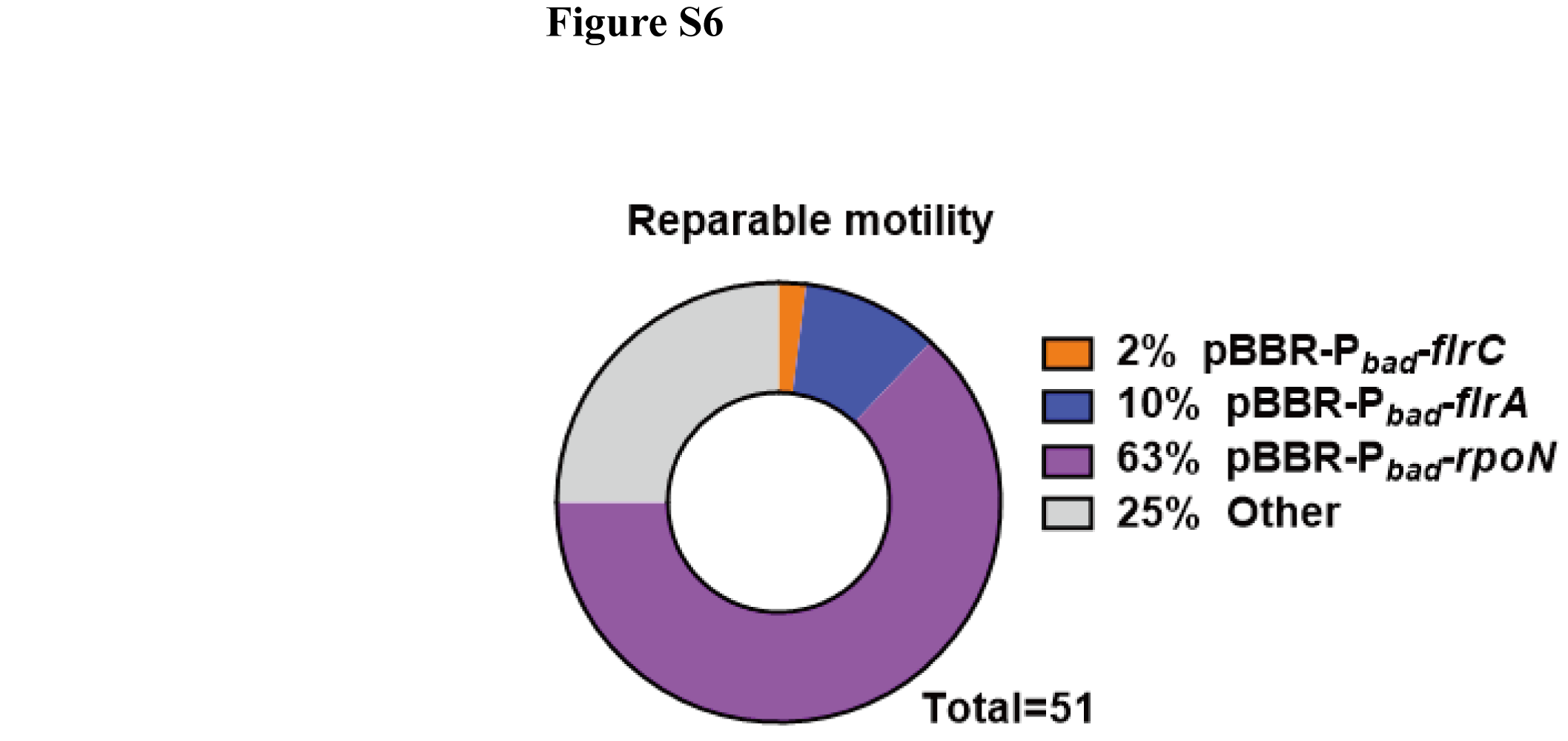

Supplement: S6 Fig — The pBBR-Pbad-flrA, pBBR-Pbad-flrC, pBBR-Pbad-rpoN plasmids were constructed to complement the motility phenotype of fifty-one nonmotile mutants derived from Δdps. Reparable motility phenotype was detected by 0.3% agar LB plates and incubated at 37°C for 8 h. (TIF) [file ppat.1011250.s006.tif]

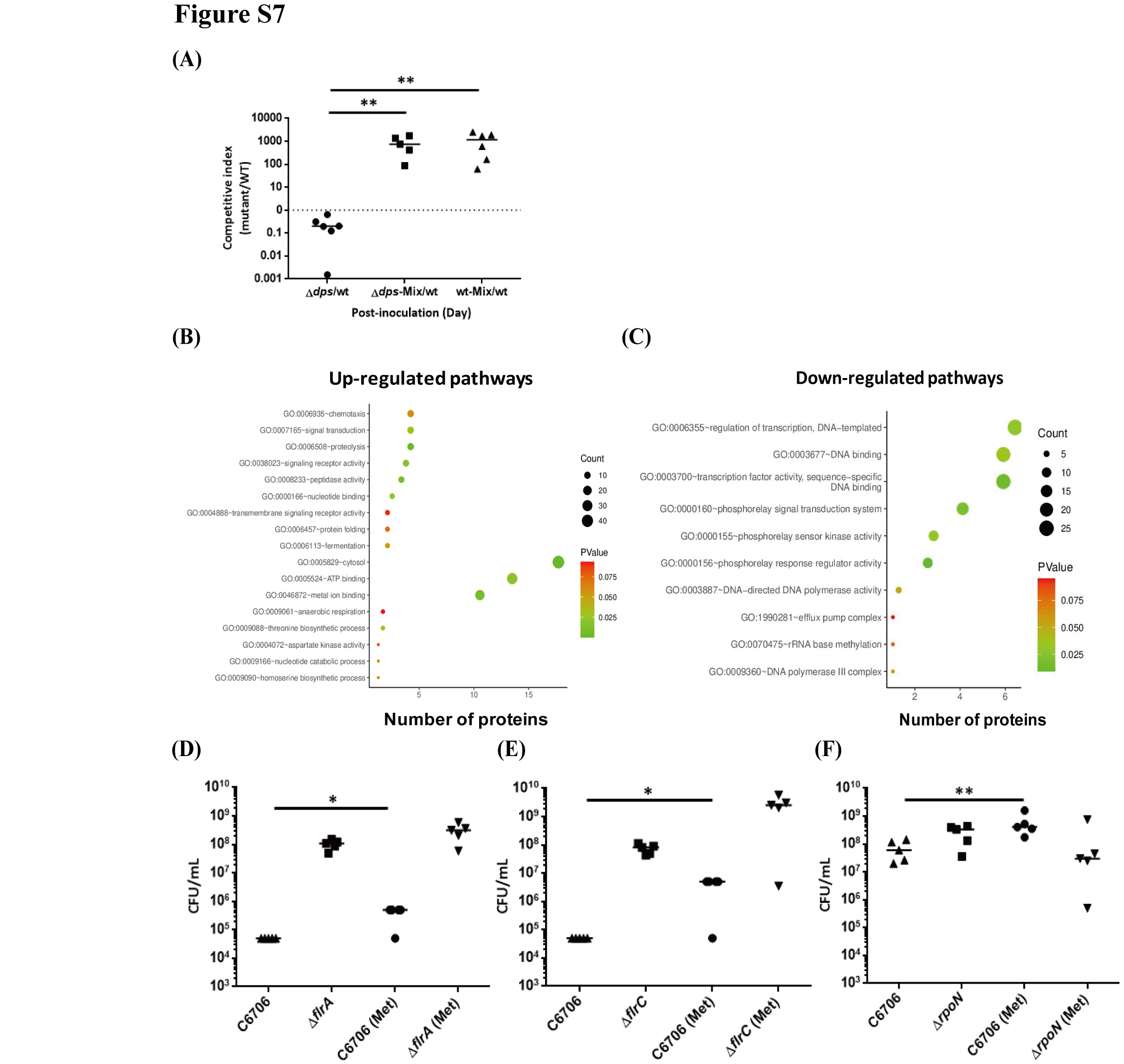

Supplement: S7 Fig — (A) Adult mice competition assay of V. cholerae from mouse intestinal filtration. We collection of V. cholerae from the feces of mice gavaged with Δdps or wild-type C6706 alone at the fifth day post-infection (as Δdps-Mix, wt-Mix), and then performed the competition colonization assay using wild-type C6706 and Δdps (left), Δdps-Mix (middle) or wt-Mix (right), the competitive index (CI) of the fifth day after infection was calculated as the ratio of mutant to wild-type colonies normalized to the input ratio. Horizontal line: median CI. Significance was determined by Kruskal-Wallis test, p-value: **, < 0.01. Gene Ontology (GO) enrichment analysis of up-regulated pathways (B) and down-regulated pathways (C) of proteomic data. We performed the bacterial precipitate proteomic analysis of ΔflrA and ΔflaA, which represented the increased (ΔflrA) and decreased (ΔflaA) colonization in adult mice. Up-regulated/Down-regulated pathways indicate increased/decreased expression in ΔflrA. See supplementary information for a complete list with proteomic data. Absolute colony numbers of wild-type C6706 and ΔflrA (D), ΔflrC (E), ΔrpoN (F) from adult mice competition assay with or without methionine. Methionine addition indicates adult mice supplemented with 25 mM L-methionine (Met) in drinking water. Horizontal line: median. Significance was determined by Mann Whitney test, p-value: *, < 0.05, **, < 0.01. (TIF) [file ppat.1011250.s007.tif]

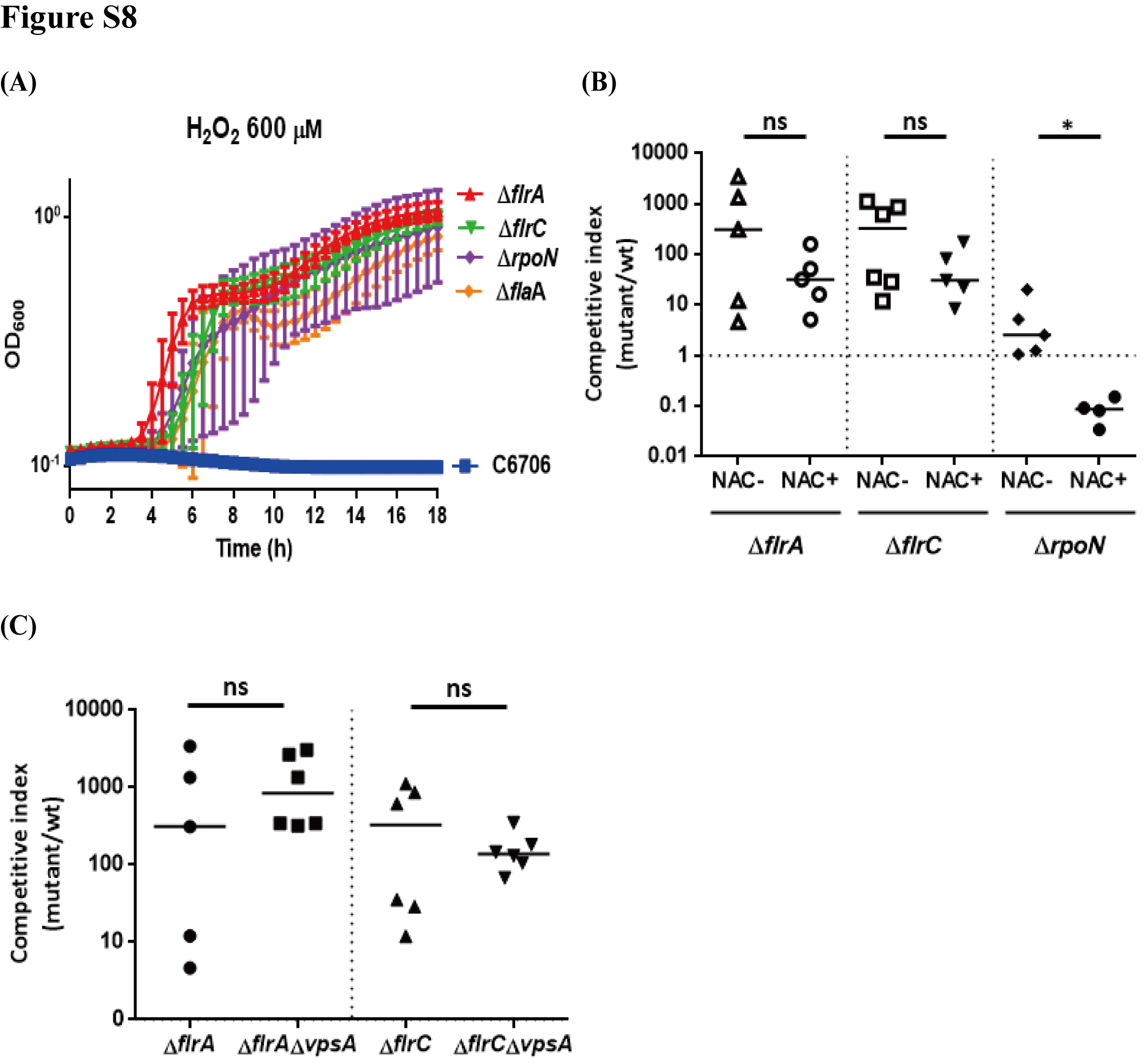

Supplement: S8 Fig — (A) Growth of wild-type and ΔflrA, ΔflrC, ΔrpoN under ROS stress. Exponentially growing cultures of wild-type C6706 (Blue), ΔflrA (red), ΔflrC (green), ΔrpoN (purple) and ΔflaA (orange) were grown in LB with 600 μM H2O2. The recovery and growth of each strains were monitored over time. The averages of 3 experiments were showed for each strain. (B) Colonization of ΔflrA, ΔflrC, ΔrpoN mutants in adult mice treated with or without NAC. 108 cells of wild-type and mutant were mixed in a 1:1 ratio and intragastrically administered to CD-1 adult mice treated with or without NAC. The competitive index (CI) of the fifth day after infection was calculated as the ratio of mutant to wild-type colonies normalized to the input ratio. Horizontal line: median CI. Significance was determined by Mann Whitney test, p-value: ns, not significant, *, < 0.05. (C) Adult mice competition assay of ΔflrAΔvpsA, ΔflrCΔvpsA. Horizontal line: median CI. Significance was determined by Mann Whitney test, p-value: ns, not significant. (TIF) [file ppat.1011250.s008.tif]

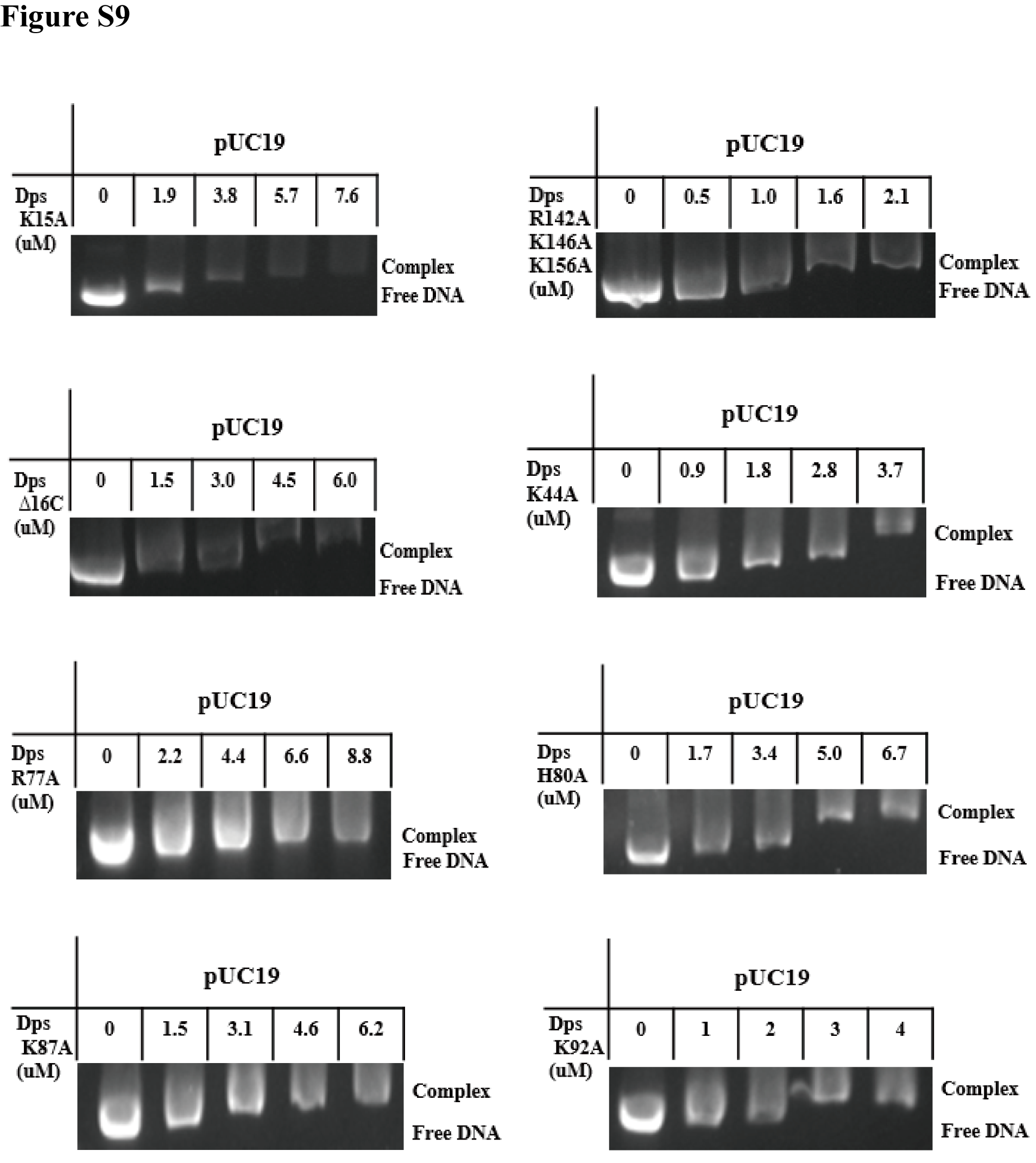

Supplement: S9 Fig — Different concentration K15A, R142AK146AK156A, Δ16C, K44A, R77A, H80A, K87A and K92A of Dps mutant protein was incubated individually with 0.6 pM of supercoiled plasmid pUC19 (in 50 mM MOPS buffer pH 7.0, containing 50 mM NaCl). (TIF) [file ppat.1011250.s009.tif]
