# Supplementary material for: Dps-dependent in vivo mutation enhances long-term host adaptation in Vibrio cholerae
Source: PLoS Pathog. 2023 Mar 16;19(3):e1011250. doi: 10.1371/journal.ppat.1011250 (PMC10104298; doi:10.1371/journal.ppat.1011250)
Supplement: S1 Table — (DOCX) [file ppat.1011250.s010.docx]

| S1 Table. Strains and plasmids used in this study. | | |
| --- | --- | --- |
| **Strains** | **Description** | **Source or reference** |
| ***V. cholerae*** |  |  |
| parent | *Vibrio cholerae* O1 El Tor C6706, wild type, Sm^R^ | [1] |
| ∆*lacZ* | C6706 Sm^R^, *lacZ* knockout | [2] |
| ∆*flaA* | C6706 Sm^R^, *flaA* knockout | Lab stock |
| ∆*flrA* | C6706 Sm^R^, *flrA* knockout | This study |
| ∆*flrC* | C6706 Sm^R^, *flrC* knockout | This study |
| ∆*rpoN* | C6706 Sm^R^, *rpoN* knockout | This study |
| ∆*mshQ* | C6706 Sm^R^, *mshQ* knockout | This study |
| ∆*tagE* | C6706 Sm^R^, *tagE* knockout | This study |
| ∆*dps* | C6706 Sm^R^, *dps* knockout | This study |
| ∆*katG* | C6706 Sm^R^, *katG* knockout | [3] |
| ∆*prxA* | C6706 Sm^R^, *prxA* knockout | This study |
| ∆*hmpA* | C6706 Sm^R^, *hmpA* knockout | This study |
| ∆*mutS* | C6706 Sm^R^, *mutS* knockout | This study |
| ∆*nfo* | C6706 Sm^R^, *nfo* knockout | This study |
| ∆*xthA* | C6706 Sm^R^, *xthA* knockout | This study |
| ∆*uvrA* | C6706 Sm^R^, *uvrA* knockout | This study |
| *flrA*-24 | C6706 Sm^R^, *flrA*1384_1385dupG | This study |
| *flrA*-48 | C6706 Sm^R^, *flrA*389delA | This study |
| *flrC*-6 | C6706 Sm^R^, *flrC*720_722delAGC | This study |
| *flrC*-18 | C6706 Sm^R^, *flrC*370_388delAAGTCAGATGACAACGGCG | This study |
| *rpoN*-47 | C6706 Sm^R^, *rpoN*762_763insA | This study |
| *dps*^D65A^ | C6706 Sm^R^, DpsD65A | This study |
| *dps*^F46E^ | C6706 Sm^R^, DpsF46E | This study |
| ∆4∆*flrA* | C6706 Sm^R^, *metRmetImetTmsrCflrA* knockout | This study |
| ∆4∆*flrC* | C6706 Sm^R^, *metRmetImetTmsrCflrC* knockout | This study |
| ∆4∆*rpoN* | C6706 Sm^R^, *metRmetImetTmsrCrpoN* knockout | This study |
| ∆*flrA*∆*vpsA* | C6706 Sm^R^, *flrAvpsA* knockout | This study |
| ∆*flrC*∆*vpsA* | C6706 Sm^R^, *flrCvpsA* knockout | This study |
| ***E. coli*** |  |  |
| DH5α λpir | Cloning host,  *supE44* Δ*lac*U169 (Φ*lacZ*ΔM15) *recA1 endA1 hsdR17 thi-1 gyrA96 relA1 λpir* | [4] |
| Sm10 λpir | Cloning host, Km^R^, *thi thr leu tonA lacY supE recA*::RP4-2-Tc::Mu (λ pirR6K) | [5] |
| Bl21 (DE3) | Expression host, F- *ompT hsdS_B_* (r_B_- m_B_-) *gal dcm* (DE3) | Lab stock |
| **Plasmids** | **Description** | **Source or reference** |
| pWM91 | Ap^R^, suicide vector for allelic exchange, *mob*^+^ | [6] |
| pWM-*flaA*-KO | Ap^R^, suicide vector for *flaA* deletion | This study |
| pWM-*flrA*-KO | Ap^R^, suicide vector for *flrA* deletion | This study |
| pWM-*flrC*-KO | Ap^R^, suicide vector for *flrC* deletion | This study |
| pWM-*rpoN*-KO | Ap^R^, suicide vector for *rpoN* deletion | This study |
| pWM-*mshQ*-KO | Ap^R^, suicide vector for *mshQ* deletion | This study |
| pWM-*tagE*-KO | Ap^R^, suicide vector for *tagE* deletion | This study |
| pWM-*dps*-KO | Ap^R^, suicide vector for *dps* deletion | This study |
| pWM-*dps* | Ap^R^, suicide vector for complementation of ∆*dps* | This study |
| pWM-*prxA*-KO | Ap^R^, suicide vector for *prxA* deletion | This study |
| pWM-*hmpA*-KO | Ap^R^, suicide vector for *hmpA* deletion | This study |
| pWM-*mutS*-KO | Ap^R^, suicide vector for *mutS* deletion | This study |
| pWM-*nfo*-KO | Ap^R^, suicide vector for *nfo* deletion | This study |
| pWM-*xthA*-KO | Ap^R^, suicide vector for *xthA* deletion | This study |
| pWM-*vurA*-KO | Ap^R^, suicide vector for *vurA* deletion | This study |
| pWM-*flrA*-24 | Ap^R^, suicide vector for *flrA*1384_1385dupG | This study |
| pWM-*flrA*-48 | Ap^R^, suicide vector for *flrA*389delA | This study |
| pWM-*flrC*-6 | Ap^R^, suicide vector for *flrC*720_722delAGC | This study |
| pWM-*flrC*-18 | Ap^R^, suicide vector for *flrC*370_388delAAGTCAGATGACAACGGCG | This study |
| pWM-*rpoN*-47 | Ap^R^, suicide vector for *rpoN*762_763insA | This study |
| pWM-*dps*^D65A^ | Ap^R^, suicide vector for DpsD65A | This study |
| pWM-*dps*^F46E^ | Ap^R^, suicide vector for DpsF46E | This study |
| pWM-*metR*-KO | Ap^R^, suicide vector for *metR* deletion | This study |
| pWM-*metI*-KO | Ap^R^, suicide vector for *metI* deletion | This study |
| pWM-*metT*-KO | Ap^R^, suicide vector for *metT* deletion | This study |
| pWM-*msrC*-KO | Ap^R^, suicide vector for *msrC* deletion | This study |
| pWM-*vpsA*-KO | Ap^R^, suicide vector for *vpsA* deletion | This study |
| pET32a | Ap^R^, Expression vector | Lab stock |
| pET32a-*dps*^D65A^ | Ap^R^, Expression vector for DpsD65A | This study |
| pET32a-*dps*^F46E^ | Ap^R^, Expression vector for DpsF46E | This study |
| pET32a-*dps*^K15A^ | Ap^R^, Expression vector for DpsK15A | This study |
| pET32a-*dps*^R142AK146AK156A^ | Ap^R^, Expression vector for DpsR142AK146AK156A | This study |
| pET32a-*dps*∆16C | Ap^R^, Expression vector for Dps∆16C | This study |
| pET32a-*dps*^K44A^ | Ap^R^, Expression vector for DpsK44A | This study |
| pET32a-*dps*^R77A^ | Ap^R^, Expression vector for DpsR77A | This study |
| pET32a-*dps*^H80A^ | Ap^R^, Expression vector for DpsH80A | This study |
| pET32a-*dps*^K87A^ | Ap^R^, Expression vector for DpsK87A | This study |
| pET32a-*dps*^K92A^ | Ap^R^, Expression vector for DpsK92A | This study |
| pBBRlux | Cm^R^, Expression vector | [7] |
| pBBR-P*_bad_*-*flrA* | Cm^R^, Overexpression of *flrA* | This study |
| pBBR-P*_bad_*-*flrC* | Cm^R^, Overexpression of *flrC* | This study |
| pBBR-P*_bad_*-*rpoN* | Cm^R^, Overexpression of *rpoN* | This study |
| pACYC177 | Km^R^, Expression vector | [8] |
| pACYC-P*_bad_*-*dps* | Km^R^, Overexpression of *dps*, pACYC177 with P_bad_ promoter inserted at the *bla* gene | This study |
| pACYC-P*_bad_*-*katG* | Km^R^, Overexpression of *katG*, pACYC177 with P_bad_ promoter inserted at the *bla* gene | This study |
| pACYC-P*_bad_*-*prxA* | Km^R^, Overexpression of *prxA*, pACYC177 with P_bad_ promoter inserted at the *bla* gene | This study |
| pACYC-P*_bad_*-*hmpA* | Km^R^, Overexpression of *hmpA*, pACYC177 with P_bad_ promoter inserted at the *bla* gene | This study |
| pACYC-P*_bad_*-*mutS* | Km^R^, Overexpression of *mutS*, pACYC177 with P_bad_ promoter inserted at the *bla* gene | This study |
| pACYC-P*_bad_*-*nfo* | Km^R^, Overexpression of *nfo*, pACYC177 with P_bad_ promoter inserted at the *bla* gene | This study |
| pACYC-P*_bad_*-*xthA* | Km^R^, Overexpression of *xthA*, pACYC177 with P_bad_ promoter inserted at the *bla* gene | This study |
| pACYC-P*_bad_*-*uvrA* | Km^R^, Overexpression of *uvrA*, pACYC177 with P_bad_ promoter inserted at the *bla* gene | This study |
| pJL1 | Ap^R^, Suicide vector for allele exchange in *V. cholerae* *lacZ* | [9] |
| pJL1-*flrA* | Ap^R^, pJL1 with P*_lac_*-*flrA* | This study |
| pJL1-*flrC* | Ap^R^, pJL1 with P*_lac_*-*flrC* | This study |
| pJL1-*rpoN* | Ap^R^, pJL1 with P*_lac_*-*rpoN* | This study |
| pUC19 | Ap^R^, supercoiled plasmid | [10] |

**References**:

1. Joelsson A, Liu Z, Zhu J. Genetic and phenotypic diversity of quorum-sensing systems in clinical and environmental isolates of Vibrio cholerae. Infection and immunity. 2006;74(2):1141-7. Epub 2006/01/24. doi: 10.1128/iai.74.2.1141-1147.2006. PubMed PMID: 16428762; PubMed Central PMCID: PMCPMC1360356.

2. Zhu J, Miller MB, Vance RE, Dziejman M, Bassler BL, Mekalanos JJ. Quorum-sensing regulators control virulence gene expression in Vibrio cholerae. Proceedings of the National Academy of Sciences of the United States of America. 2002;99(5):3129-34. Epub 2002/02/21. doi: 10.1073/pnas.052694299. PubMed PMID: 11854465; PubMed Central PMCID: PMCPMC122484.

3. Ma Y, Yang X, Wang H, Qin Z, Yi C, Shi C, et al. CBS-derived H2S facilitates host colonization of Vibrio cholerae by promoting the iron-dependent catalase activity of KatB. PLoS pathogens. 2021;17(7):e1009763. Epub 2021/07/21. doi: 10.1371/journal.ppat.1009763. PubMed PMID: 34283874; PubMed Central PMCID: PMCPMC8324212.

4. Hanahan D. Studies on transformation of Escherichia coli with plasmids. Journal of molecular biology. 1983;166(4):557-80. Epub 1983/06/05. doi: 10.1016/s0022-2836(83)80284-8. PubMed PMID: 6345791.

5. Miller VL, Mekalanos JJ. A novel suicide vector and its use in construction of insertion mutations: osmoregulation of outer membrane proteins and virulence determinants in Vibrio cholerae requires toxR. Journal of bacteriology. 1988;170(6):2575-83. Epub 1988/06/01. doi: 10.1128/jb.170.6.2575-2583.1988. PubMed PMID: 2836362; PubMed Central PMCID: PMCPMC211174.

6. Metcalf WW, Jiang W, Daniels LL, Kim SK, Haldimann A, Wanner BL. Conditionally replicative and conjugative plasmids carrying lacZ alpha for cloning, mutagenesis, and allele replacement in bacteria. Plasmid. 1996;35(1):1-13. Epub 1996/01/01. doi: 10.1006/plas.1996.0001. PubMed PMID: 8693022.

7. Hammer BK, Bassler BL. Regulatory small RNAs circumvent the conventional quorum sensing pathway in pandemic Vibrio cholerae. Proceedings of the National Academy of Sciences of the United States of America. 2007;104(27):11145-9. Epub 2007/06/09. doi: 10.1073/pnas.0703860104. PubMed PMID: 17556542; PubMed Central PMCID: PMCPMC1888797.

8. Chang AC, Cohen SN. Construction and characterization of amplifiable multicopy DNA cloning vehicles derived from the P15A cryptic miniplasmid. Journal of bacteriology. 1978;134(3):1141-56. Epub 1978/06/01. doi: 10.1128/jb.134.3.1141-1156.1978. PubMed PMID: 149110; PubMed Central PMCID: PMCPMC222365.

9. Liu Z, Yang M, Peterfreund GL, Tsou AM, Selamoglu N, Daldal F, et al. Vibrio cholerae anaerobic induction of virulence gene expression is controlled by thiol-based switches of virulence regulator AphB. Proceedings of the National Academy of Sciences of the United States of America. 2011;108(2):810-5. Epub 2010/12/29. doi: 10.1073/pnas.1014640108. PubMed PMID: 21187377; PubMed Central PMCID: PMCPMC3021084.

10. Norrander J, Kempe T, Messing J. Construction of improved M13 vectors using oligodeoxynucleotide-directed mutagenesis. Gene. 1983;26(1):101-6. Epub 1983/12/01. doi: 10.1016/0378-1119(83)90040-9. PubMed PMID: 6323249.
